# *trithorax* is an essential regulator of cardiac *Hox* gene expression and anterior-posterior patterning of the *Drosophila* embryonic heart tube

**DOI:** 10.1242/bio.061919

**Published:** 2025-04-02

**Authors:** Adam J. Farmer, Rajnandani Katariya, Sumaiya Islam, Md. Sayeed Abu Rayhan, Mark H. Inlow, Shaad M. Ahmad, Kristopher R. Schwab

**Affiliations:** ^1^ Department of Biology, Indiana State University, Terre Haute, IN 47809, USA; ^2^ The Rich and Robin Porter Cancer Research Center, Indiana State University, Terre Haute, IN 47809, USA; ^3^ The Center for Genomic Advocacy, Indiana State University, Terre Haute, IN 47809, USA; ^4^ Department of Mathematics and Computer Science, Indiana State University, Terre Haute, IN 47809, USA

**Keywords:** *Trithorax*, Hox, *Abdominal A*, Heart patterning, Cardiac, *Drosophila*

## Abstract

The precise regulation of transcription required for embryonic development is partially controlled by the actions of the Trithorax group (TrxG) and Polycomb group (PcG) proteins. The genes *trithorax* (*trx*), *trithorax-related* (*trr*), and *SET domain containing 1* (*Set1*) encode COMPASS-like histone methyltransferases, a subgroup of TrxG proteins that impart H3K4 methylation modifications onto chromatin in order to activate and maintain transcription. In this study, we identify the role of these genes in the development of the embryonic heart of the fruit fly *Drosophila melanogaster*. *trx, trr*, and *Set1* independently ensure proper cardiac cell divisions. Additionally, *trx* regulation of collinear *Hox* expression is necessary for the anterior-posterior cardiac patterning of the linear heart tube. *trx* inactivation in *Drosophila* results in a remarkable homeotic transformation of the posterior heart-proper segment into an aorta-like fate due to the loss of posterior *abdominal A* expression. Furthermore, cardiac expression of *Antennapedia*, *Ultrabithorax*, and *Abdominal B* is also deregulated in *trx* mutants. Together, these data suggest that the COMPASS-like histone methyltransferases are essential developmental regulators of cardiogenesis, being necessary for both cardiac cell divisions and heart patterning.

## Teaser

**Summary:** The COMPASS-like histone methyltransferase *trithorax* regulates proper cardiac cell division and is required for collinear *Hox* expression which patterns the anterior-posterior axis of the *Drosophila* heart tube.

## INTRODUCTION

Embryonic development requires precise transcriptional regulation which is partially controlled by the actions of the Trithorax group (TrxG) and Polycomb group (PcG) proteins. The founding member of the *Drosophila* TrxG genes, *trithorax* (*trx*), encodes a SET-domain-containing histone methyltransferase (HMT) within the COMPASS-like family of histone 3 lysine 4 (H3K4) methylation complexes (Kingston and Tamkun, 2014). The *trx* homologs, *trithorax-related* (*trr*) and *SET domain containing 1* (*Set1*), also encode HMTs and form their own unique COMPASS-like complexes. The post-translational H3K4 methylation marks deposited by these complexes serve as docking sites to recruit chromatin remodeling complexes and other regulatory machinery necessary for transcriptional activation and maintenance during development (Cheng, 2014). Several studies have identified the *trx* and *trr* mammalian orthologs, *lysine (K)-specific methyltransferase A-D* (*Kmt2a-d*, also known as *Mll1-4*), as important epigenetic regulators of mammalian embryonic development (Shilatifard, 2012). Although the COMPASS-like HMTs activate and maintain the transcription of diverse gene classes, *trx* has maintained its evolutionarily conserved role as a positive regulator of *Hox* expression. *Hox* activity is a major determinant of the anterior/posterior (A/P) patterning in both insects and mammals and *Hox* genes have essential roles in orchestrating organ development (Roux and Zaffran, 2016; Soshnikova and Duboule, 2009). Multiple *Hox* genes cooperatively regulate the contributions of cardiac progenitor cell populations to embryonic heart patterning in both invertebrate and vertebrate organisms (Deschamps and van Nes, 2005; Lescroart and Zaffran, 2018). In mammals, the inactivation of the *trx* ortholog *Kmt2b* results in severe cardiac abnormalities and early embryonic lethality, indicating that these genes are necessary for proper cardiac gene expression (Glaser et al., 2006; Goldsworthy et al., 2013). Therefore, the function of COMPASS-like HMTs in the regulation of *Hox* and other cardiac genes remains an important area of investigation in developmental biology (Wang, 2012).

In this report, we investigated the role of the COMPASS-like HMTs *trx*, *trr*, and *Set1* in cardiac development using the embryonic dorsal vessel (linear heart tube) of the fruit fly *Drosophila melanogaster*, which offered several advantages as an experimental system. The initial stages of invertebrate and vertebrate embryonic heart development share morphological, anatomical, and genetic similarities including the evolutionary conservation of a cardiac development regulatory network (Ahmad, 2017; Bodmer and Frasch, 2010; Bodmer and Venkatesh, 1998; Cripps and Olson, 2002; Meganathan et al., 2015; Olson, 2006; Tao and Schulz, 2007; Vogler and Bodmer, 2015). However, the *Drosophila* dorsal vessel develops as a single linear epithelial tube consisting of exactly 104 contractile cardial cells surrounded by a sheath of supportive and nephrocytic pericardial cells, thereby allowing the investigation of a wide-range of developmental and cell division defects at a single cell resolution. Additionally, while genetic duplication of the COMPASS-like HMTs has occurred within the mammalian genome, the *Drosophila* orthologs exist as single genes thereby reducing potentially confounding effects due to functional redundancy and compensation (Chintapalli et al., 2007; El-Brolosy and Stainier, 2017; Ocorr et al., 2007; Shilatifard, 2012).

Numerous cell division defects were identified throughout the dorsal vessel in the individual lethal mutants of *trx*, *trr*, and *Set1* indicating an independent contribution of each gene to cardiac cell division control. Furthermore, we describe a critical role for *trx* in controlling the collinear expression of *Hox* genes, which pattern the A/P axis of the dorsal vessel. *trx* inactivation induces a remarkable homeotic transformation of the posterior heart-proper segment into an aorta-like fate due to the loss of *abdominal A* (*abd-A*) expression within the posterior dorsal vessel. Additionally, *trx* is necessary for the proper cardiac expression of *Antennapedia* (*Antp*), *Ultrabithorax* (*Ubx*), and *Abdominal B* (*Abd-B*). Together, these data indicate that the COMPASS-like HMTs are essential developmental regulators of cardiogenesis necessary for both cardiac cell division and heart patterning.

## RESULTS

### The *Drosophila* COMPASS-like HMTs regulate proper cardiac cell division

**Fig. 1.**
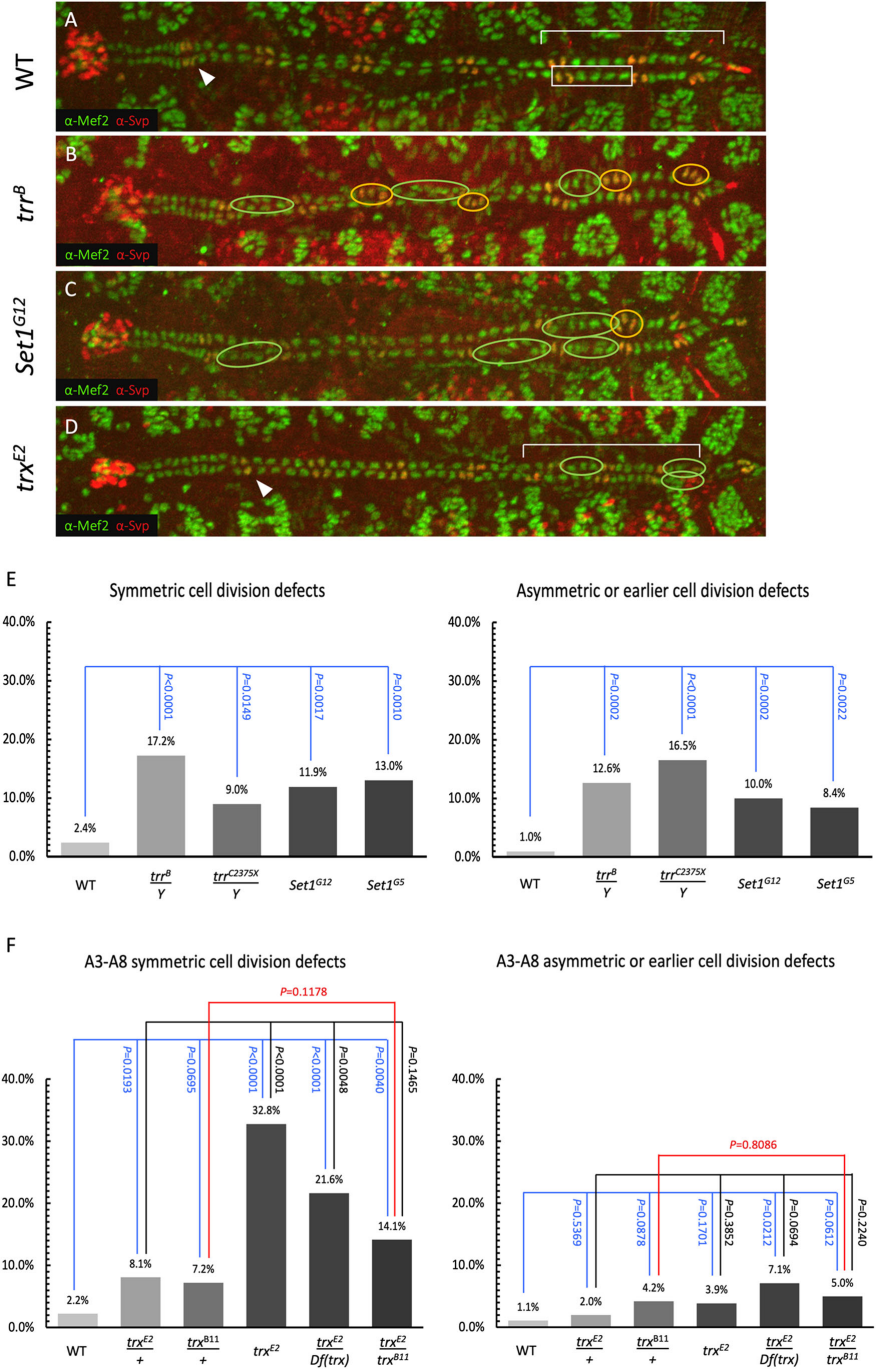
**Cardiac cell division phenotypes of H3K4 HMT mutant dorsal vessels.** (A) Mef2 and Svp immunostaining of the dorsal vessel from a stage 16 wild-type embryo identifying the Svp CCs (yellow) and Tin CCs (green). An arrowhead marks the Svp CCs in the A2 cardiac hemisegments of the posterior aorta while a bracket outlines the posterior heart-proper region. The A2-A7 cardiac hemisegments are each composed of two Svp CCs followed by four Tin CCs. An example of a hemisegment is indicated by a box outlining the left A6 heart-proper hemisegment. (B,C) The *trr^B^* (B) and *Set1^G12^* (C) mutants exhibit cell division defects within both the Tin CC (green ellipses) and Svp CC (orange ellipses) lineages disrupting the normal two Svp CC and four Tin CC hemisegment pattern. (D) Cell division defects within the Tin CC lineage (green ellipses) are detected in the A3-A8 hemisegments of the *trx^E2^* mutant embryo. The *trx* mutant displays two additional reproducible phenotypes in addition to the cell division defects. The mutant does not display the enlarged lumen of the heart-proper region (D, bracket) in the posterior dorsal vessel compared to wild type (A, bracket). The typical two Svp CCs identified in each wild-type A2 hemisegments (A, arrowhead) is consistently reduced to a single Svp CC in the *trx* mutant (D, arrowhead). (E) Fraction of hemisegments exhibiting symmetric and asymmetric or earlier cell divisions in the A2-A8 hemisegments of wild-type, *trr* mutant, and *Set1* mutant embryos. The number of embryos and hemisegments, respectively, examined for each genotype are as follows: wild type=15 and 210, *trr^B^/Y*=17 and 238, *trr^C2375X^/Y*=16 and 212, *Set1^G12^*=15 and 210, and *Set1^G5^*=15 and 210. The relative significance of each type of cell division compared with wild type is shown. (F) Fraction of hemisegments exhibiting symmetric and asymmetric or earlier cell divisions in the A3-A8 hemisegments of wild-type embryos and *trx* mutants. Whereas *trr* and *Set1* mutants exhibit both significant increases in symmetric and asymmetric or earlier cell division defects, the *trx* mutants display only a significant increase in symmetric cell division defects. The number of embryos and hemisegments, respectively, examined for each genotype are as follows: wild type=15 and 180, *trx^E2^/+*=17 and 200, *trx^B11^/+*=14 and 166, *trx^E2^*=15 and 180, *trx^E2^/Df(trx)*=17 and 196, and *trx^E2^/trx^B11^*=17 and 200. The relative significance of each type of cell division compared with wild type, *trx^E2^/+*, and *trx^B11^/+* is shown.

The *Drosophila* COMPASS-like HMTs *trx*, *trr*, and *Set1* catalyze H3K4 methylation marks on chromatin associated with poised or active transcription of important developmental regulatory genes (Shilatifard, 2012). To explore the function of COMPASS-like HMTs in cardiogenesis, we utilized the *Drosophila* dorsal vessel. The *Drosophila* dorsal vessel at embryonic stage 16 consists of a myoepithelial tube that exhibits axial symmetry, A/P patterning, and a repeated cellular pattern (Figs 1A and 6A). This structure can simply be described as a linear heart tube composed of myoepithelial cardial cells (CCs) that generate the apical lumen and create the contractile force to propel hemolymph throughout the vessel. The CCs of the abdominal A2 to A8 hemisegments are organized into repeated metameric cellular structures which are primarily derived from the *seven-up* (*svp*) and *tinman* (*tin*) cardiac progenitor lineages (Alvarez et al., 2003; Han and Bodmer, 2003; Ward and Skeath, 2000). Each cardiac hemisegment consists of a repeated pattern of two Svp CCs followed by four Tin CCs, except for A8, which is abbreviated to two Svp CCs followed by only two Tin CCs (Figs 1A and 6A). In each cardiac hemisegment, a cell division event at an earlier stage generates two Svp cardiac progenitor cells, with each Svp progenitor cell subsequently undergoing asymmetric cell division to produce a Svp CC and an associated *odd skipped*-expressing pericardial cell (Alvarez et al., 2003; Han and Bodmer, 2003; Ward and Skeath, 2000). Thus, any deviation from the expected number of two Svp CCs in a hemisegment indicates a defect in either asymmetric or earlier cell division. In contrast, in the *tin* cardiac lineage, an initial symmetric cell division produces two Tin cardiac progenitor cells, each of which subsequently divides symmetrically to produce two myocardial Tin CCs (Alvarez et al., 2003; Han and Bodmer, 2003; Ward and Skeath, 2000). Hence a total of four Tin CCs are generated in each hemisegment and any alteration in the expected number of Tin CCs reflects defective symmetric cardiac progenitor cell division (Ahmad et al., 2014, 2012; Kump et al., 2021).

*Drosophila* embryos hemizygous or homozygous for lethal H3K4 HMT alleles of *trr^B^*, *Set1^G12^*, and *trx^E2^* were evaluated for cardiac cell division defects using this strategy. Svp and Tin CC numbers in individual hemisegments in these HMT mutants and wild-type embryos were assessed and compared by immunostaining with either anti-H15 (Neuromancer1, Nmr1) and anti-Seven up (Svp) antibodies (which labeled all CCs and exclusively Svp CCs, respectively) or anti-Myocyte enhancer factor 2 (Mef2) and anti-Svp antibodies (which labeled all CCs and exclusively Svp CCs, respectively) (Fig. 1A-D). To quantify cardiac cell division defects, the A2 to A8 hemisegments of homozygous *trr^B^* or *Set1^G12^* mutant embryos were examined and scored for an increase or decrease of Tin CCs, corresponding to symmetric cell division defects, or Svp CCs, indicating earlier symmetric and/or later asymmetric cell division defects, compared to wild type. Our investigation found that embryos homozygous for either *trr^B^* or *Set1^G12^* mutations exhibited a significant increase over wild type in the fraction of hemisegments exhibiting cell division defects for both the Tin and Svp lineages (Fig. 1A-C,E, Table S1). To confirm that these cardiac phenotypes were indeed a consequence of loss or reduction of *trr* or *Set1* functions, embryos hemizygous and homozygous for the *trr^C2375X^* and *Set1^G5^* alleles, respectively, were also evaluated for changes in CC numbers. Similarly significant increases over wild type in cell division defects were also obtained for these two alleles, demonstrating that *trr* and *Set1* were essential for mediating proper cardiac cell divisons (Fig. 1E, Figs S1G-J and S2D-K, Table S1).

**Fig. 2.**
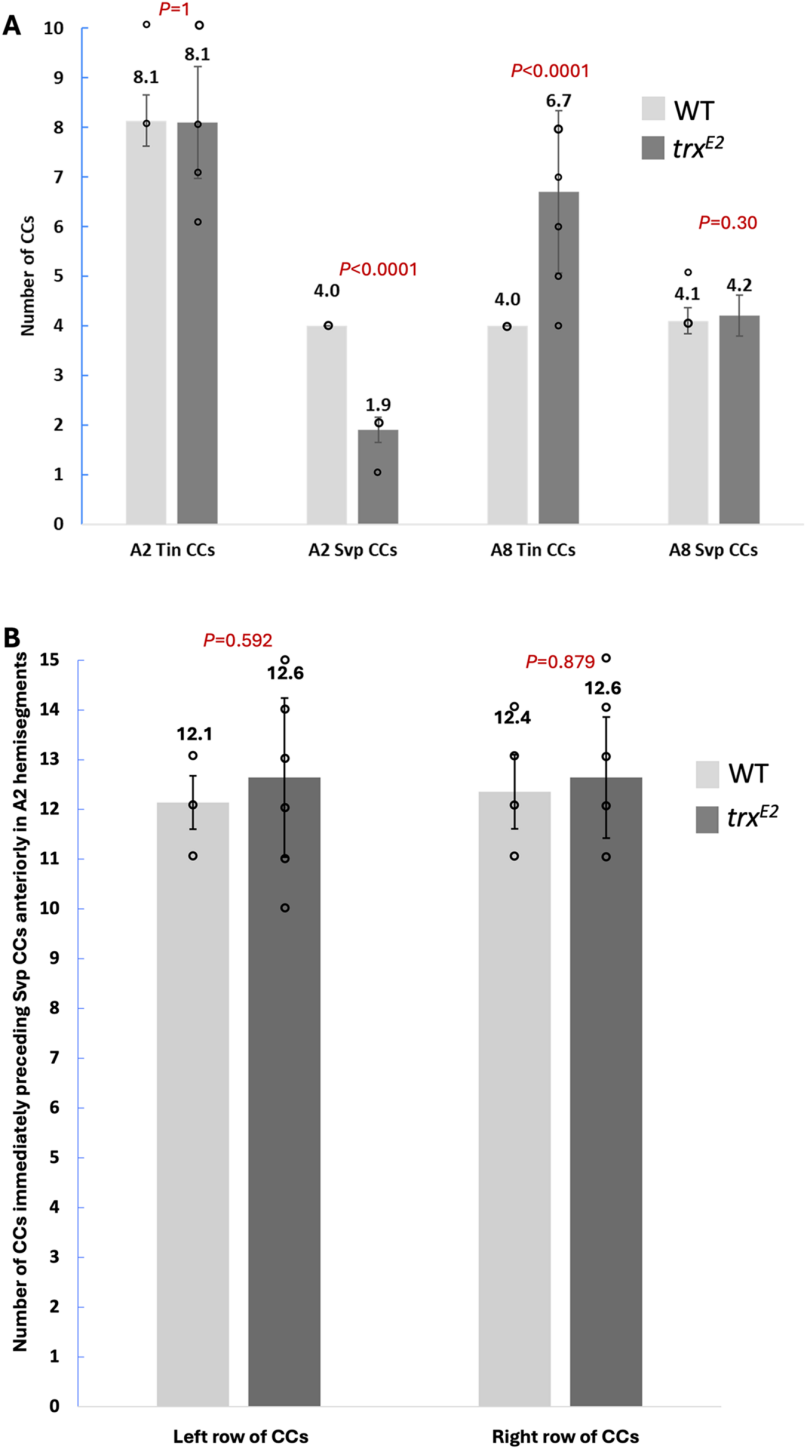
**Quantitation and comparison of relevant cardial cell (CC) numbers in wild**-**type and *trx* mutant embryos.** (A) Comparison of Tin CC and Svp CC numbers in A2 and A8 segments in wild-type embryos and *trx^E2^* mutants. Each wild-type A2 segment, comprised of two contralateral A2 hemisegments, consists of approximately eight Tin CCs and four Svp CCs (i.e. four Tin CCs and two Svp CCs per hemisegment). While the number of Tin CCs is not affected in the A2 segments of *trx* mutant embryos, the number of Svp CCs is reduced by half (i.e. each A2 hemisegment in *trx* mutant embryos contains one Svp-CC in place of the expected two of wild-type embryos). These results indicate a defect in the cardiac progenitor cell division process that gives rise to two Svp-CCs in the A2 hemisegments in *trx* mutants. Each wild-type A8 segment consists of approximately four Tin CCs and four Svp-CCs (i.e. each of the two posterior most contralateral A8 hemisegments comprising this segment have two Tin-CCs and 2 Svp-CCs). While the number of Svp-CCs in the A8 segment is not significantly altered in *trx* mutant embryos, the number of Tin-CCs is significantly increased over that in wild-type embryos. These results demonstrate that *trx* serves to restrict Tin CC progenitor differentiation in the A8 segment. 15 segments (30 hemisegments) were analyzed for segment specific CC numbers for each genotype. Mean CC numbers±s.d. are reported. For quantitation of Tin CC and Svp CC number changes in A2 and A8 hemisegments, two-tailed, two-sample unequal variance *t*-tests were used. (B) Comparison of CCs immediately preceding the Svp CCs of the A2 hemisegments anteriorly in wild-type embryos and *trx^E2^* mutants. The number of these (approximately 12 for each row) CCs are not significantly different between the two genotypes, thereby indicating that the single Svp CC in A2 hemisegments of *trx* mutants are not a consequence of one of the two expected Svp CCs failing to express Svp. Left and right rows for 14 embryos of each genotype were analyzed. Mean CC numbers±s.d. are reported. One-way ANOVAs were used for comparing these genotypes.

The *trx^E2^* homozygous mutant embryos, however, exhibited an intriguing, completely penetrant A2 hemisegment-specific phenotype. The expected two Svp CCs in each A2 hemisegment in the wild-type dorsal vessel were reduced to a single Svp CC in each and every one of the 30 *trx* mutant A2 hemisegments we examined (Fig. 1A,D, arrowheads, Fig. 2A, Fig. S2A). While we did observe changes in Svp CC number in A2 hemisegments in the other HMT mutants, they were not anywhere near as penetrant (4/30 for *trr^B^* mutants and 7/30 for *Set1^G12^* mutants compared to 0/30 for wild-type embryos). Unlike the *trx* mutants, where the Svp CC number in the A2 hemisegments was consistently reduced to one, *trr* and *Set1* mutants also exhibited A2 hemisegments with more than two Svp CCs compared to wild type. To verify that this cardiac phenotype was due to disruption of *trx* function, and not a consequence of a mutation at a second site, transheterozygous embryos of the genotypes *trx^E2^/trx^B11^* (where *trx^B11^* is an amorphic *trx* allele) and *trx^E2^/Df(3R)BSC470* (where the *Df(3R)BSC470* deficiency, henceforth referred to as *Df(trx)*, completely deletes the *trx* gene) were evaluated in comparison with the individual *trx^E2^/+* and *trx^B11^/+* heterozygotes. Homozygous *trx^B11^* stage 16 embryos were not recoverable due to likely early embryonic lethality. The reduction of two Svp CCs to one in the A2 hemisegments in the *trx^E2^* mutants (Fig. 2) was also observed in the *trx^E2^*/*trx^B11^* and *trx^E2^/Df(trx)* transheterozygous embryos (Figs S1D-F and S3A).

Two distinct hypotheses could explain this result. The cell destined to be the anteriormost Svp CC in the A2 hemisegment in the wild-type embryo could simply fail to express detectable Svp protein in the *trx* mutant. An alternative explanation is a missing Svp CC in the A2 hemisegment in *trx* mutant embryos as a consequence of either an asymmetric or earlier cell division defect. The Svp CCs in each A2 hemisegment in the wild-type embryo are immediately preceded anteriorly by the 12 CCs of the anterior aorta that do not express Svp. Hence, if the first hypothesis, that of the anteriormost Svp CC of the A2 hemisegment failing to express Svp in *trx* mutants is correct, then the single Svp CC in the A2 hemisegment would be immediately preceded by 13 CCs that do not express Svp. In contrast, if the second hypothesis is correct, that a cell division defect prevents the development of more than one Svp CC in the A2 hemisegments of *trx* mutants, then the sole Svp CC in the mutant hemisegment and the two Svp CCs in wild-type A2 hemisegment would both be immediately preceded anteriorly by the same number of CCs, the 12 cells of the anterior aorta lacking Svp expression. Our quantification of the CCs immediately preceding the Svp CC(s) in A2 hemisegments revealed no statistically significant difference in cell number between wild-type embryos and *trx^E2^* mutants in either the left or the right row of the embryonic heart (the left row displayed an average of 12.1 CCs in wild-type embryos compared to the mutant's average of 12.6 CCs, *P*=0.592; while the wild type right row exhibited an average of 12.4 CCs compared to the *trx* mutant average of 12.6 CCs, *P*=0.879) (Fig. 2B). These results indicate that the precursors of the Svp lineage in A2 hemisegments of embryos lacking *trx* function did not reproducibly complete the stereotypical cell divisions to generate two Svp CCs (Alvarez et al., 2003; Gajewski et al., 2000; Han and Bodmer, 2003; Ward and Skeath, 2000).

Because *trx* mutants exhibited this additional completely penetrant A2 hemisegment-specific alteration in Svp CC number, we were concerned that incorporating data for the A2 hemisegments could potentially contaminate and skew our statistical assessment of cell division defects in the more posterior cardiac hemisegments. In order to eliminate any such influence or bias, we compared hemisegments A3 to A8 exclusively between embryos lacking *trx* function, embryos heterozygous for *trx* mutants, and wild-type embryos for changes in Tin CC and Svp CC numbers. Our results showed that *trx* mutants also exhibited significantly more symmetric cell division defects (indicated by more or fewer Tin CCs) compared to wild-type embryos in hemisegments A3 to A8 (Fig. 1F, Figs S1 and S2, Table S1). However, in contrast to our observations with *trr* and *Set1* mutants, we found that the fraction of A3 to A8 hemisegments exhibiting changes in the expected Svp CC number in *trx^E2^* mutants was not significantly different from that in wild type embryos (Fig. 1F, Table S1). Similarly, the fraction of A3 to A8 hemisegments exhibiting changes in the Svp CC number in the trans-heterozygous *trx^E2^/Df(trx)* embryos was not significantly different from that in heterozygous *trx^E2^/+* embryos, and the fraction in *trx^E2^*/*trx^B11^* was not significantly different from that in either *trx^E2^/+* or *trx^B11^/+* embryos (Fig. 1F, Fig. S1, Table S1). Thus, in the posterior A3 to A8 hemisegments, *trx* is essential for mediating symmetric cardiac cell divisions, but is not required for either asymmetric cardiac progenitor cell divisions, or for the cell divisions at an earlier stage that give rise to a pair of Svp progenitors.

### *trx* is essential for the dilated lumen of the posterior region of the dorsal vessel

The *Drosophila* dorsal vessel is patterned into two anatomical areas along the A/P axis: the posterior heart-proper region (hemisegments A6 to A8), which absorbs fluid from the posterior embryo and propels hemolymph into the aorta, and the aorta (hemisegments T2 to A5), which transports hemolymph to the anterior embryo (Fig. 6A). The heart-proper contains columnar CCs with elliptical nuclei, which surround an enlarged lumen, while the long aorta consists of cuboidal CCs, which generate a narrow lumen. The aorta itself can be further subdivided into two regions based upon the presence of Svp CCs. The anterior aorta (hemisegments T2 to A1) is devoid of Svp CCs, while the posterior aorta (hemisegments A2 to A5) and heart-proper are both organized into hemisegments containing both Svp CCs and Tin CCs. However, the Svp CCs in the posterior aorta can be distinguished from those in the heart-proper since only the latter differentiate into valvular inlet ostia cells as a result of both *svp* and *abd-A* activity (Curtis et al., 1999; Lo et al., 2002; Lovato et al., 2002; Molina and Cripps, 2001; Ponzielli et al., 2002).

**Fig. 3.**
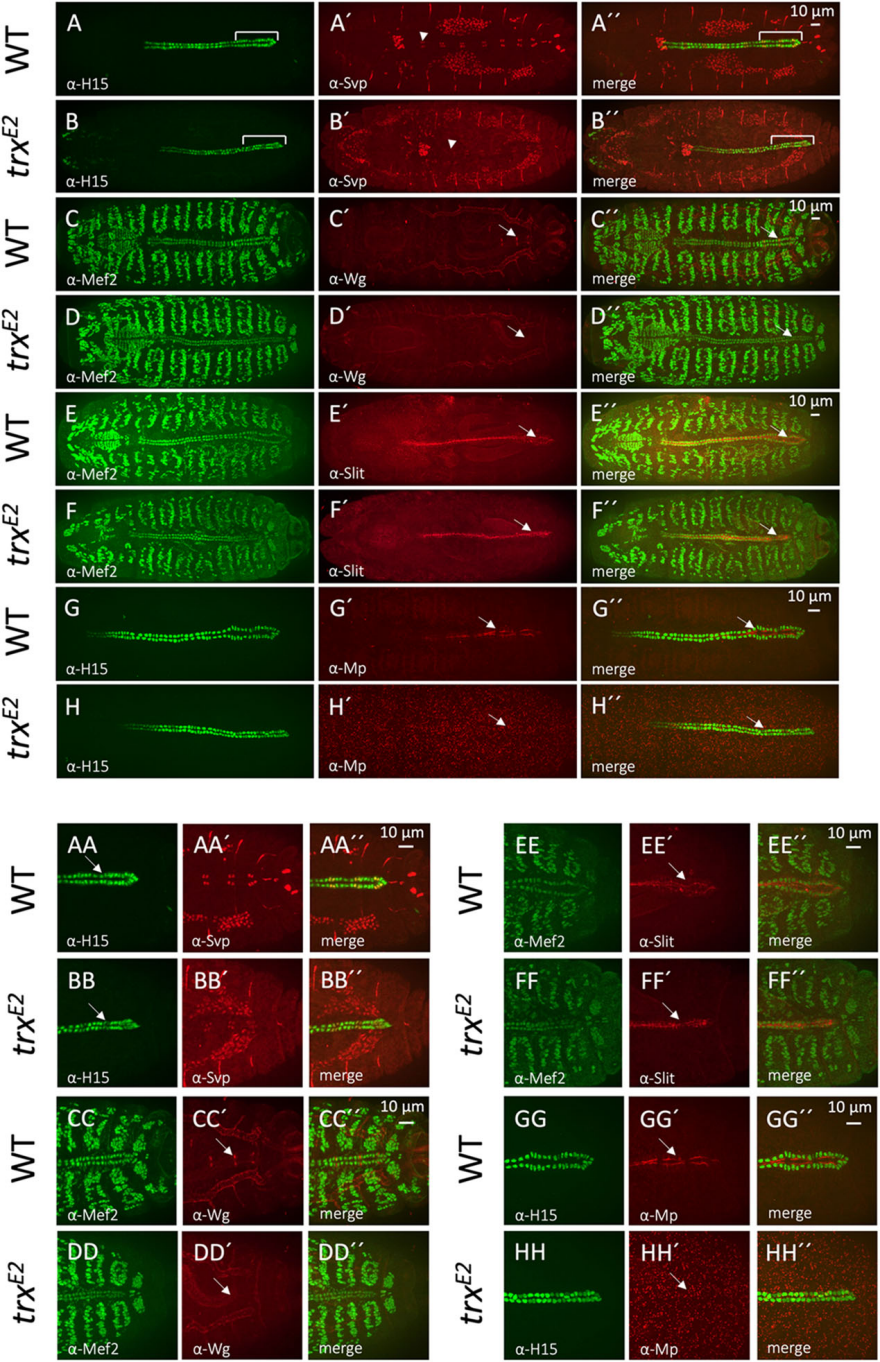
**Morphology and cardiac patterning within wild**-**type and *trx* mutant embryos.** (A-B″) Svp CC patterning in wild-type and *trx^E2^* mutant dorsal vessels. All CCs were labeled using anti-H15 (green) and Svp CCs using anti-Svp (red, yellow in merge). The wild-type embryo (A-A″) exhibits two Svp CCs in each hemisegment beginning with the A2 hemisegment (A′, arrowhead) and the characteristic heart-proper dilation (A, A″ bracket, *n*=15, 15/15). The *trx* mutant (B-B″) maintains the expected Svp CC expression pattern although the overall levels of Svp protein are reduced (*n*=15, 15/15). In addition, note that the number of Svp CCs in the *trx* mutant A2 hemisegments is reduced to one (B′, arrowhead) and an absence of dilation within the posterior heart-proper region (B, B″, bracket). (AA-BB″) Higher magnification views of the heart proper region within images A-A″ and B-B″. Note the posterior luminal reduction in the *trx* mutant (BB, arrow) compared to wild type (AA, arrow). (C-D″) Valvular ostial cells are visualized within the wild-type and *trx^E2^* heart-proper region using anti-Mef2 (green) and anti-Wg (red). The three pairs of Svp ostial cells located within the wild-type heart-proper (C′ and C″, arrow) exhibit high levels of Wg staining (*n*=5, 5/5). However, Wg protein is completely lost from the *trx* posterior dorsal vessel (D′ and D″, arrow) indicating an absence of ostial cell development with the posterior dorsal vessel (*n*=4, 4/4). (CC-DD″) Higher magnification views of the heart proper region within images C-C″ and D-D″. Note the absence of Wg immunostaining within the *trx* mutant (DD′, arrow) compared to wild type (CC′, arrow). (E-H″) Investigation of epithelialization and aorta luminal formation within the wild-type and *trx^E2^* dorsal vessels using the cardiac luminal anti-Slit (red) and anti-Mef2 (green) immunostaining. In both the wild-type (E-E″, *n*=11, 11/11) and *trx* mutant (F-F″, *n*=7, 7/7) embryos, similar levels of Slit immunostaining are localized within the lumen along the entire length of the dorsal vessel, suggesting that epithelization and aorta formation are not significantly affected in the *trx* mutant. The heart-proper region is identified by an arrow. (EE-FF″) Higher magnification views of the heart-proper region within images E-E″ and F-F″. Note that Slit immunostaining persists within the heart-proper region of both the wild type (EE′, arrow) and *trx* mutant (FF′, arrow). (G-H″) Posterior luminal patterning within the wild-type and *trx^E2^* mutant dorsal vessels is visualized using the luminal collagen marker anti-Mp (red) and anti-Mef2 (green). Within the wild-type embryo (G-G″), Mp is localized to the posterior dorsal vessel and functions in heart-proper lumen formation and enlargement (G′ and G″, arrow, *n*=5, 5/5). Strikingly, Mp within the *trx* mutant (H-H″) is completely absent from the dorsal vessel (H′ and H″, arrow, *n*=4, 4/4), confirming the transformation of the heart-proper into an aorta-like fate. (GG-HH″) Higher magnification views of the heart-proper region within images G-G″ and H-H″. Note that Mp immunostaining strongly stains the wild-type heart-proper region (GG′, arrow), but is completely absent within the *trx* mutant (HH′, arrow).

While the *trr* mutant and *Set1* mutant embryos exhibited a dilated lumen in the heart-proper region similar to that of wild-type embryos (Fig. 1A-C), embryos lacking *trx* function exhibited heart-proper regions that resembled the narrow-luminal, cuboidal epithelial appearance of the posterior aorta (Fig. 1D). The average lumen width of the posterior aorta and heart-proper region within the wild type is 0.66 µm and 2.18 µm, respectively (Fig. S4). Specifically, our analysis revealed that while the mean width of the lumen of the heart-proper regions was significantly greater than that of the posterior aorta in wild-type, *trr* mutant and *Set1* mutant embryos (*P*<0.0001 for wild type, *P*=0.0021 and *P*<0.0001 for *trr^B^/Y* and *trr^C2375X^/Y* genotypes, respectively, and *P*<0.0001 for either *Set1^G12^* or *Set1^G5^* genotypes), there was no significant difference in the width of the lumen between these two regions in embryos of the genotypes *trx^E2^* (*P*=0.1976), *trx^E2^/Df(trx)* (*P*=0.0601), and *trx^E2^*/*trx^B11^* (*P*=0.1453) (Fig. S4). To better visualize the morphology of the dorsal vessel, wild-type and *trx* mutant embryos were immunostained using antibodies directed against Svp and H15, a cardiac T-box transcription factor with expression restricted to the CC lineage (Qian et al., 2005a). Confirming our results in Fig. 1D, we identified the aorta-like morphology of the heart-proper region within *trx* mutant embryos (Fig. 3A-B″,AA-BB″). Additionally, closer inspection revealed another significant discrepancy between the CCs in wild-type and *trx* mutant embryos: although the Svp CCs are readily identifiable within *trx* mutants, the Svp CCs of the dorsal vessel in embryos lacking *trx* function express reproducibly lower levels of Svp protein compared to those in wild-type controls (Fig. S3A′,B′,AA′,BB′). Similar reductions in Svp protein levels were also identified within the *trx^E2^*/*trx^B11^* and *trx^E2^/Df(trx)* mutants (Fig. S1E′,F′).

### *trx* maintains *wg* expression in the Svp CCs of the heart-proper region

Within the A6-A8 hemisegments of the heart-proper, both *svp* and *abd-A* are necessary for the induction of *wingless* (*wg*) expression within the Svp cardiac progenitors and the differentiation of these cells into the valvular ostial cells (Lo et al., 2002; Ponzielli et al., 2002; Trujillo et al., 2016). Consequently, in wild-type embryos, the Svp CCs of the heart-proper (hemisegments A6 to A8) are distinguishable from the Svp CCs in the posterior aorta (hemisegments A2 to A5) since the former express both *svp* and *wg* while the latter express *svp* alone.

The narrow-luminal aorta-like morphology of the heart-proper region in embryos lacking *trx* function that we observed phenocopies that of *abd-A* mutant embryos (Lo et al., 2002; Lovato et al., 2002; Perrin et al., 2004; Ryan et al., 2005) and suggests that the posterior dorsal vessel in *trx* mutant homozygotes may have undergone a homeotic transformation and adopted the anterior fate of the aorta. If this is true, then the Svp CCs in the homeotically transformed heart-proper region of *trx* mutant embryos would express only *svp*, not both *svp* and *wg* (Lo et al., 2002; Perrin et al., 2004). While Wg immunostaining readily identifies the Svp CC ostial pairs within the wild-type embryo, Wg protein is completely lost within the *trx* mutant embryo and coincides with the absence of the expected ostial cell morphology (Fig. 3C-D″,CC-DD″, arrows). Therefore, the heart-proper region is indeed homeotically transformed in embryos lacking *trx* function in a manner which phenocopies the loss of *wg*-expressing ostial cells within *abd-A* mutants.

### *trx* is necessary for the expression of the extracellular protein Multiplexin in the posterior dorsal vessel

Several extracellular proteins necessary for the development and maintenance of the cardiac lumen exhibit either uniform or differential expression within the *trx* dorsal vessel. Whereas *slit* expression is necessary for cardiac luminal development and is localized uniformly across the entire myoepithelia of dorsal vessel (Fig. 3E-E″,EE-EE″), high expression of *Multiplexin* (*Mp*) is required for the wider heart-proper luminal dilation and is localized to a more posterior domain (Harpaz et al., 2013; MacMullin and Jacobs, 2006; Qian et al., 2005b; Santiago-Martínez et al., 2006; Volk et al., 2014). Specifically, a gradient of Mp is detected within the wild-type dorsal vessel with levels precipitously declining along the posterior aorta in a posterior-to-anterior manner until the anterior region is devoid of signal (Fig. 3G-G″,GG-GG″). Slit immunostaining reveals similar levels of expression within both wild-type and *trx* mutant embryos suggesting that aorta lumen formation and epithelialization may not be significantly affected by *trx* loss of function (Fig. 3E-F″,EE-FF″). However, Mp immunostaining reveals a dramatic absence of this protein within the *trx* mutant dorsal vessel compared to that in the wild-type control consistent with a complete loss of heart-proper specification (Fig. 3G-H″,GG-HH″). Collectively, these data and the observations described in the previous sections identify several heart-proper and posterior dorsal vessel patterning defects within the *trx* mutant and confirm a homeotic transformation of the heart-proper into a posterior aorta-like fate.

### *trx* specifies the heart-proper fate by activating abd-A expression

**Fig. 4.**
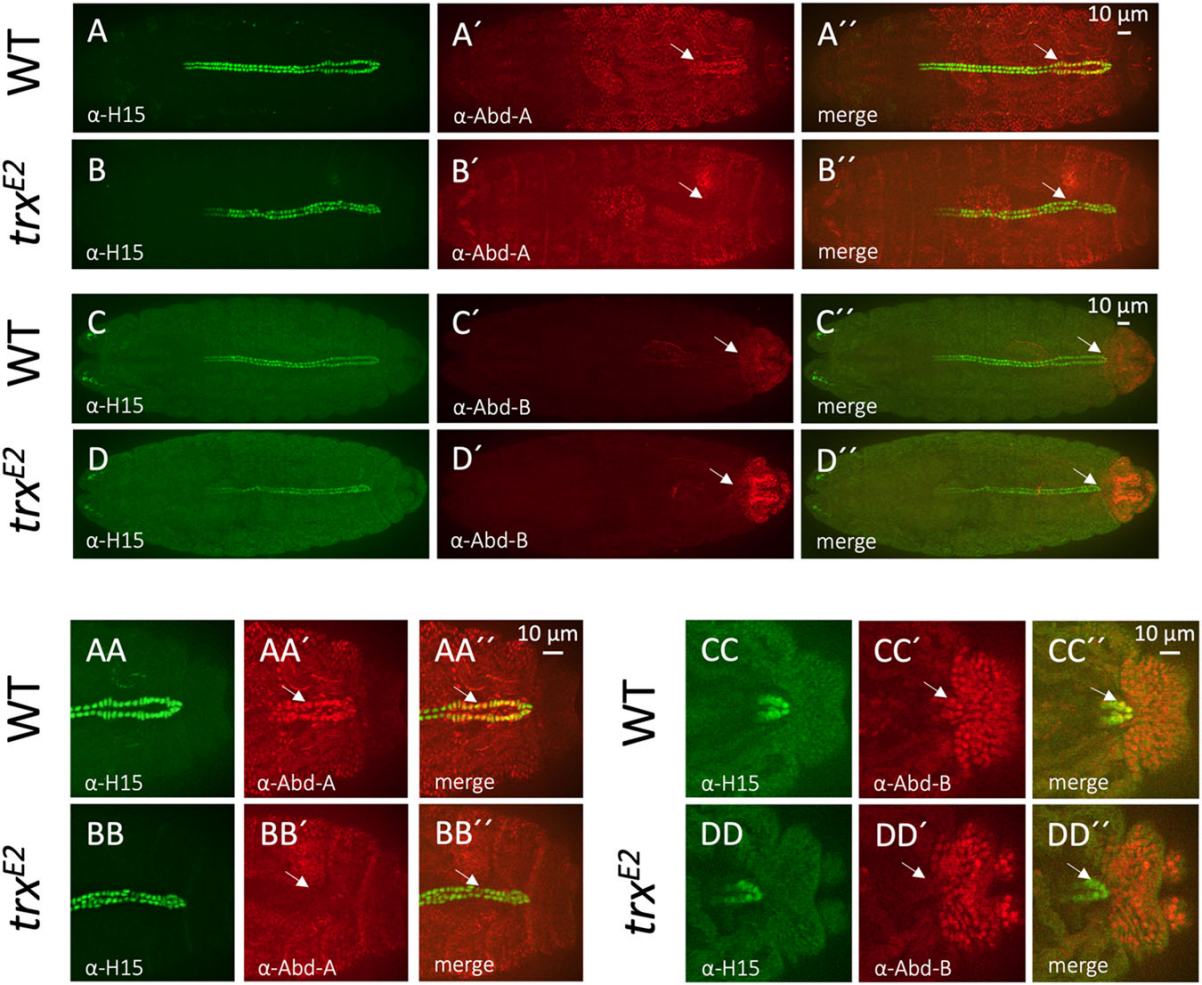
**Loss of heart-proper-specific abdominal *Hox* gene expression within the *trx* mutant embryos.** *Abd-A* or *Abd-B* expression (red) within wild-type and *trx^E2^* mutant dorsal vessels is detected by antibody staining. All CCs of the dorsal vessel are identified using anti-H15 (green). (A-B″) The heart-proper region of the wild-type embryo (A′ and A″, arrow) displays strong Abd-A immunostaining (*n*=5, 5/5 embryos), while the posterior *trx* mutant dorsal vessel (B′ and B″, arrow) lacks any Abd-A staining (*n*=4, 4/4 embryos). (AA-BB″) Higher magnification views of the heart-proper regions in A-B″. The heart-proper region of the wild-type embryo (AA′ and AA″, arrow) displays strong Abd-A immunostaining, while the posterior *trx* mutant dorsal vessel (BB′ and BB″, arrow) lacks any Abd-A staining. These results indicate that the homeotic transformation of the *trx* mutant posterior dorsal vessel is due to loss of Abd-A, the primary selector of the heart-proper region. (C-D″) Intense Abd-B immunostaining marks the posterior most aspect of the wild-type heart-proper region (C′ and C″, arrow, *n*=6, 6/6 embryos). However, the Abd-B protein is completely lost within posterior Tin CCs of the *trx* mutant (D′ and D″, arrow, *n*=7, 7/7 embryos). (CC-DD″) Higher magnification view of the heart-proper region. Note the posterior CCs of the wild-type heart-proper region (CC′ and CC″, arrow) displays high Abd-B immunostaining, while the posterior CCs of the *trx* mutant (DD′ and DD″, arrow) are devoid of Abd-B.

**Fig. 5.**
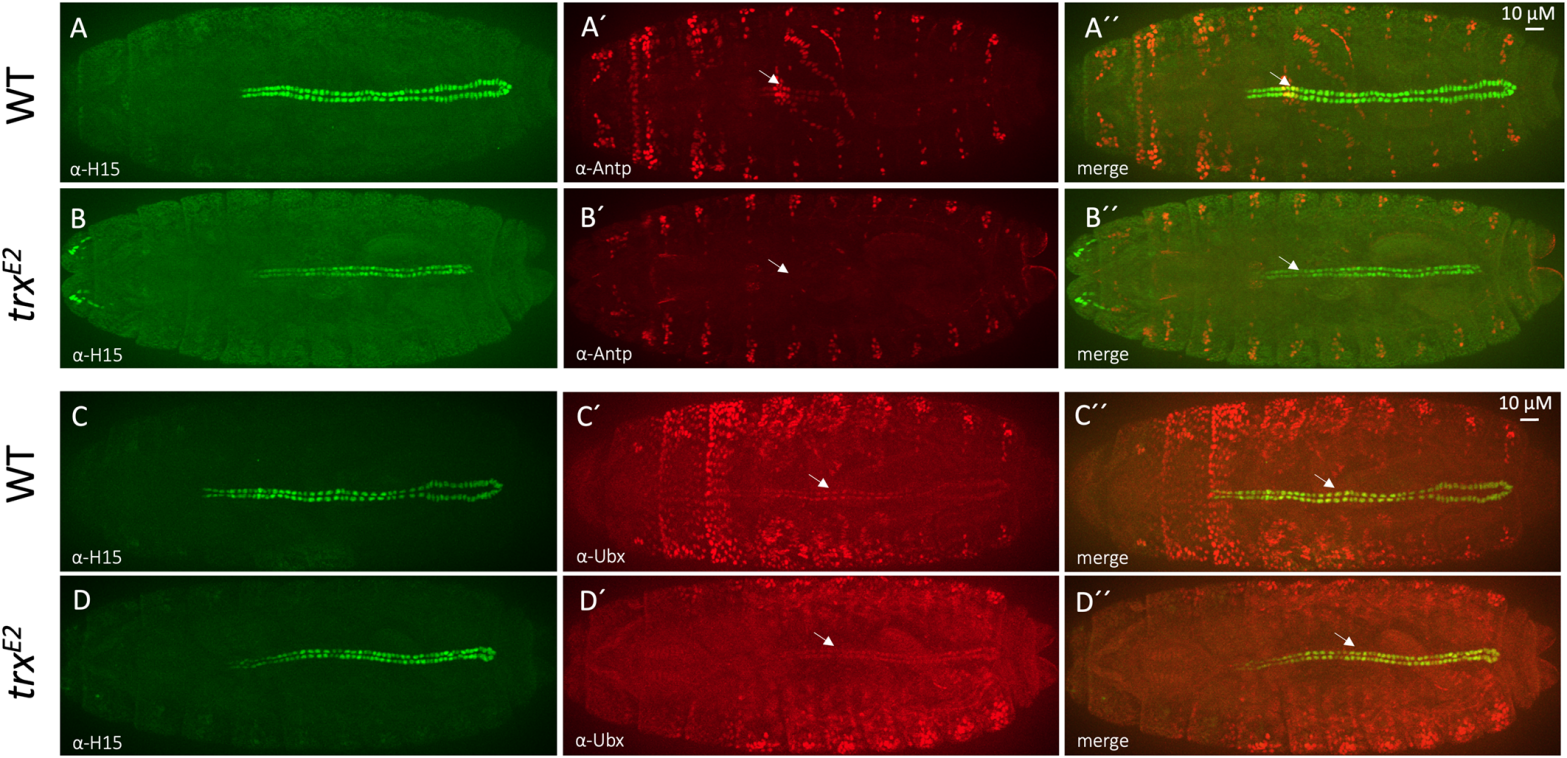
**Aortic *Antp* and *Ubx* expression within the *trx* mutant dorsal vessel.** *Antp* or *Ubx* expression domains in wild-type and *trx^E2^* mutant embryos are detected by immunostaining with the appropriate antibodies (red), while all CCs of the dorsal vessel are identified using the anti-H15 antibody (green). (A-B″) Antp protein demarks the boundary between the anterior and posterior aorta. (A-A″) High *Antp* expression within the wild-type dorsal vessel (*n*=11, 11/11) is located at the boundary between the anterior and posterior aorta (A′, arrow). (B-B″) In contrast, *Antp* expression is completely lost within the aorta of the *trx* mutant embryo (B′, arrow, *n*=8, 8/8). (C-D″) Ubx protein is broadly localized across the posterior aorta. (C-C″) In wild-type embryos, *Ubx* is expressed at different levels along the dorsal vessel, being highest across the A2-A4 hemisegments of the posterior aorta (C′ and C″, arrow) and declining where it overlaps the *Antp* and *abd-A* expression domains (*n*=6, 6/6). (D-D″) In the *trx* mutant dorsal vessel, *Ubx* expression persists, albeit with an altered expression pattern (*n*=8, 8/8). The *Ubx* expression levels within the A2-A4 hemisegments (B′,B″, arrow) are reduced such that a uniform and consistent level of *Ubx* expression is maintained throughout the posterior dorsal vessel in the *trx* mutant.

**Fig. 6.**
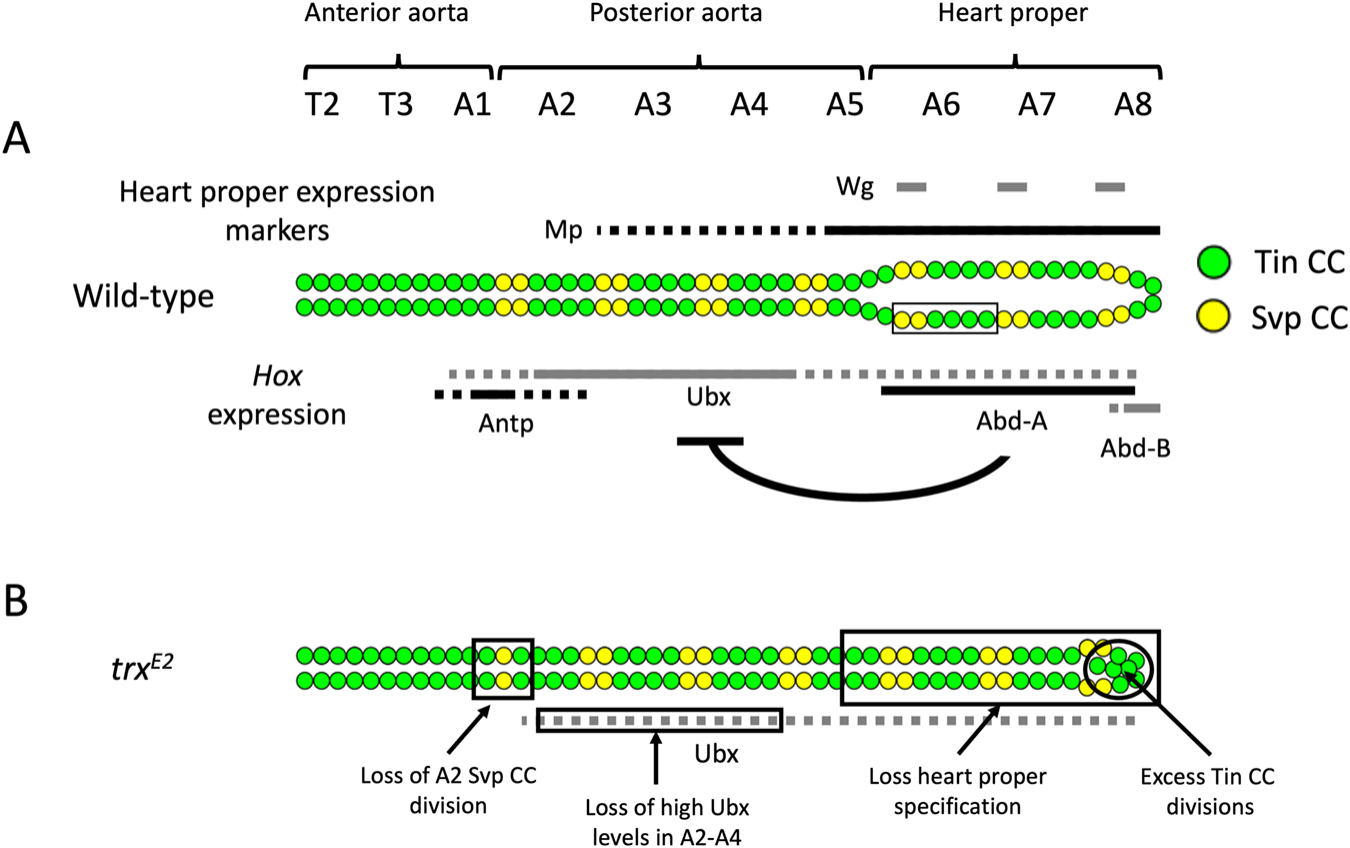
**Morphology of the wild**-**type and *trx* mutant dorsal vessels.** (A) The wild-type dorsal vessel is organized into three regions: the anterior aorta, posterior aorta, and heart-proper. The posterior aorta and heart-proper exhibit the repeated arrangement of two pairs of Svp cardiac cells (CCs) (yellow) followed by four pairs of Tin CCs (green), which comprise a cardiac hemisegment (box). The heart-proper is distinguished by its enlarged lumen, the differentiation of Svp CCs into valvular ostial cells, and an abbreviated posterior most contralateral pair of A8 hemisegments consisting of two Svp CCs and two Tin CCs each. The expression domains of the posterior dorsal vessel markers *wg* and *Mp* are indicated above the wild-type dorsal vessel, while the expression domains of *Antp*, *Ubx*, *abd-A*, and *Abd-B* are presented below. High expression is indicated by a solid line while low expression is identified by a dashed line. *Abd-A* mediated repression of *Ubx* is also indicated. (B) The *trx* mutant dorsal vessel phenotypes are summarized along with the changes in *Hox* expression and cardiac patterning. *Antp*, *abd-A*, and *Abd-B* expression is lost within the dorsal vessel in *trx* mutant embryos. However, *Ubx* expression is reduced to a uniform and consistent level throughout the posterior dorsal vessel. The most striking phenotypic feature of the *trx* dorsal vessel is the homeotic transformation of heart-proper into a posterior aorta-like fate due to the loss of Abd-A activity. Additionally, the loss of anterior Antp and posterior Abd-B activity within the dorsal vessel results in the reduction of Svp cardiac progenitor cell division in the A2 hemisegments and the derepression of Tin cardiac progenitor cell differentiation in the A8 hemisegments, respectively.

*Drosophila trx* and its vertebrate orthologs partially regulate the collinear *Hox* selector gene expressions that in turn pattern the A/P axis of the embryo (Breen and Harte, 1991, 1993; Hanson et al., 1999; Ingham, 1998; Yu et al., 1995). Although the knowledge of TrxG regulation of *Hox* activity in embryo patterning is well understood, *trx* regulation of cardiac-specific *Hox* expression in heart development remains an open area of investigation. Fig. 6A summarizes the collinear expression of the *Antennapedia* (*Antp*) and *Bithorax complex* (*Bx-C*) *Hox* genes *Ubx*, *abd-A*, and *Abd-B* across A/P axis of the dorsal vessel in wild-type embryos (Lo and Frasch, 2003; Lovato and Cripps, 2016; Monier et al., 2007). *Hox* inputs play an additional role in Svp CC progenitor specification across this axis (Perrin et al., 2004; Ryan et al., 2005). Together with our observations that the posterior dorsal vessel is homeotically transformed into the aorta in *trx* mutants in a manner reminiscent of *abd-A* mutants, these data suggest that *trx* may control cardiac fate along the A/P axis by regulating collinear *Hox* expression. If this is true, then the expression of the *Hox* genes would be expected to be significantly altered in *trx* mutants. Thus, to investigate *Hox* expression within the dorsal vessel, stage 16 *trx* mutant and wild-type embryos were immunostained with a panel of antibodies directed against the known *Hox* genes expressed within the dorsal vessel (Figs 4 and 5, Figs S5, S6, S7).

Previous studies have identified *abd-A* as the primary *Hox* selector gene responsible for specifying the heart-proper region (Lo et al., 2002; Lovato et al., 2002; Ponzielli et al., 2002). In the wild-type control, Abd-A immunostaining identifies high levels of expression within the A5-A8 segments that form the heart-proper (Fig. 4A-A″,AA-AA″, arrows). As predicted from the loss of heart-proper specification, immunostaining in the *trx^E2^* mutants reveals a complete loss of *abd-A* expression within the posterior dorsal vessel (Fig. 4B-B″,BB-BB″ compared to Fig. 4A-A″, AA-AA″, arrows). Similar results were obtained for the *trx^E2^/trx^B11^* and *trx^E2^/Df(trx)* transheterozygous mutants compared to *trx^E2^*/+ and *trx^B11^*/+ heterozygotes (Fig. S5C-D″,CC-DD″ compared to Fig. S5A-B″,AA-BB″, arrows). Furthermore, these results replicate the previously reported loss of cardiac *abd-A* expression of the amorphic *trx^B11^* strain (Breen and Harte, 1993). Since the loss of *trx* function abrogates *abd-A* expression and phenocopies the *abd-A* mutant cardiac phenotype, the loss of *abd-A* expression in the *trx* mutant is likely the cause of the heart-proper to aorta transformation.

### *trx* restricts the number of Tin CCs in the A8 hemisegments by activating *Abd-B* expression

Next, we evaluated cardiac *Abd-B* expression, which is restricted to the posterior-most A8 segment of the wild-type dorsal vessel (Fig. 4C-C″,CC-CC″, arrows). Previous reports indicate that *Abd-B* activity suppresses the formation of the cardiac mesodermal lineage within the posterior-most parasegments of the wild-type embryo (Lo et al., 2002; Lovato et al., 2002; Ponzielli et al., 2002). Although heart-proper formation is not affected in the *Abd-B* mutants, the posterior most A8 hemisegment of the dorsal vessel is populated by supernumerary CCs, which may be derived from the loss of *Abd-B* repression of cardiac mesoderm specification within parasegment 13 (Lo et al., 2002). We found that *Abd-B* expression is completely lost from the A8 cardiac segment within the *trx* mutant in contrast to wild-type embryos (Fig. 4D-D″,DD-DD″ compared to Fig. 4C-C″,CC-CC″, arrows). This result was also replicated in the *trx^E2^/trx^B11^* and *trx^E2^/Df(trx)* transheterozygous mutants compared to *trx^E2^*/+ and *trx^B11^*/+ heterozygotes (Fig. S6C-D″,CC-DD″ compared to Fig. S6A-B″,AA-BB″, arrows).

To investigate whether the *trx* mutants also phenocopy the supernumerary CC defect in *Abd-B* mutants, the number of CCs within the A8 segment (comprised of both contralateral A8 hemisegments) of the wild-type and *trx^E2^* mutant dorsal vessels were quantified. This analysis revealed a statistically significant increase within the Tin CC population of the *trx^E2^* mutant (6.7 CCs) compared to the wild type (4.0 CCs); in contrast, the number of Svp CCs in the A8 hemisegments did not exhibit any significant variation between wild-type and *trx* mutant embryos (Fig. 2A). Analysis of the A8 Svp and Tin CCs identified similar results within the *trx^E2^/trx^B11^* (6.1 CCs) and *trx^E2^/Df(trx)* (5.2 CCs) transheterozygous mutants compared to *trx^E2^*/+ (4.1 CCs) and *trx^B11^*/+ (3.9 CCs) heterozygotes (Fig. S3B). Thus, *trx* functions to restrict Tin CC progenitor differentiation in these posteriormost cardiac hemisegments by activating *Abd-B* expression in the posterior embryonic segments.

Collectively, the findings reported in this, and the previous section identify a critical role for *trx* in maintaining cardiac *abd-A* and *Abd-B* expression within the heart-proper region.

### *trx* mediates cardiac cell division along the Svp lineage in the A2 segment by activating *Antp* expression

The cardiac *Antp* activity facilitates the generation of two Svp CC pairs within the A2 segment of the dorsal vessel (Perrin et al., 2004; Ryan et al., 2005). Interestingly, the individual *Antp* and *trx* mutants share a common A2 phenotype: the expected two pairs of Svp CCs in the A2 segments are found to be reduced to a single pair (Fig. 1A compared to Figs 1D and 3A′ compared to Fig. 3B′, arrowheads, Fig. S1D-F″ compared to Fig. S1A-C″). Given this similar phenotype, it is likely that *trx* may regulate cardiac cell divisions along the Svp lineage in A2 by controlling *Antp* expression. Therefore, Antp immunostaining was performed for both wild-type and *trx* mutant embryos to assess potential *Antp* expression changes (Fig. 5A-B″, Fig. S7A-D″). The *Antp* expression domain within the wild-type embryo is located at the A1/A2 boundary, which separates the anterior and posterior regions of the aorta (Fig. 5A-A″, arrows). In the *trx* mutant, *Antp* is completely lost within the anterior dorsal vessel, thereby indicating that *trx* activity is indeed required for cardiac *Antp* expression (Fig. 5B-B″ compared to Fig. 5A-A″, arrows). Evaluation of the *trx^E2^/trx^B11^* and *trx^E2^/Df(trx)* transheterozygous mutants revealed a similar loss of Antp protein at the A1/A2 boundary (Fig. S7C-D″ compared to Fig. S7A-B″).

To investigate whether the reduction of A2 Svp CC pairs was a reproducible cell division defect within the *trx* mutant, the Svp and Tin CCs of the A2 segments were quantified in both wild-type embryos and *trx* mutants. This analysis revealed a statistically significant reduction of Svp CCs in the A2 segment from a mean of 4.0 in wild-type embryos to a mean of 1.9 in the *trx^E2^* mutants. In contrast, the number of A2 Tin CCs were not significantly changed between wild-type and *trx^E2^* mutant embryos (Fig. 2A). Assessment of the *trx^E2^/trx^B11^* and *trx^E2^/Df(trx)* transheterozygous mutants identified similar results (Fig. S3A). As previously mentioned, the number of CCs immediately anterior to the Svp CCs in the wild-type and *trx^E2^* mutant A2 segments exhibited no significant difference (Fig. 2B), suggesting that *Antp* is necessary for proper Svp lineage cell division rather than *Svp* expression. These data indicate that *trx* is required for *Antp* expression and subsequent A2 segment-specific cell division in the Svp lineage in a manner similar to that of the *Hox* gene *Antp*. The most parsimonious explanation for these observations is that *trx* activates *Antp* expression to mediate proper cardiac cell division in the Svp lineage of the A2 hemisegments.

### *trx* maintains distinct expression levels of *Ubx* along the dorsal vessel

Posterior to the *Antp* expression domain, *Ubx* activity within the posterior aorta specifies the Svp CCs of the A3-A5 segments (Ryan et al., 2005). Interestingly, *Ubx* is expressed at different levels along the dorsal vessel in wild-type embryos, being highest across the A2-A4 hemisegments of the posterior aorta and declining where it overlaps the *Antp* and *abd-A* expression domains (Fig. 5C-C″) (Perrin et al., 2004). In contrast to the other *Hox* genes we observed whose expression is eliminated in embryos lacking *trx* function, *Ubx* expression persists within the *trx* mutant, but its expression pattern is significantly altered (Fig. 5D-D″ compared to Fig. 5C-C″). The noticeably higher levels of *Ubx* expression within the A2-A4 hemisegments in wild-type embryos are reduced such that a uniform and consistent level of *Ubx* is expressed throughout the dorsal vessel in *trx* mutants (Fig. 5D-D″, arrows). Interestingly, Ubx immunostaining of the *trx^E2^/trx^B11^* and *trx^E2^/Df(trx)* transheterozygous mutants identified similarly lower protein levels within the posterior heart tube (Fig. S7E-H″).

Together, the collinear expression of *Antp*, *Ubx*, and *abd-A* within their corresponding domains is necessary to specify the Svp progenitor lineage and ensure the proper cell division of these Svp progenitors since the loss of all *Hox* function results in a complete loss of Svp CCs (Perrin et al., 2004). The continued expression of *Ubx* combined with the absence of *Antp* and *abd-A* expression within the *trx* mutant dorsal vessel suggests that this remaining *Ubx* activity is sufficient to maintain Svp CC lineage specification and cell divisions throughout the dorsal vessel except for the Svp CCs located within A2 hemisegments (Fig. 1D compared to Figs 1A and 3B-B″ compared to Fig. 3A-A″, Fig. S1D-F″ compared to Fig. S1A-C″). However, the expression of merely *Ubx* in the posterior dorsal vessel in *trx* mutants is insufficient to drive heart-proper specification, consistent with prior studies identifying the *abd-A* function as the primary selector for this process (Lo et al., 2002; Lovato et al., 2002; Perrin et al., 2004; Ponzielli et al., 2002).

## DISCUSSION

In addition to their roles of important transcriptional co-activators, the *trx*, *trr*, and *Set1* genes encode essential HMTs of unique COMPASS-like complexes that catalyze H3K4 methylation, an important chromatin modification associated with transcription (Cheng, 2014; Kingston and Tamkun, 2014; Shilatifard, 2012). Our study reveals that each of these genes contributes to the regulation of Svp and Tin cardiac cell divisions necessary for the cellular organization of the embryonic dorsal vessel. We show further that one of these three HMTs, *trx*, patterns the cardiac A/P axis by maintaining the proper collinear expression of *Hox* genes.

Although this study describes the zygotic role of these genes in cardiac cell division and patterning, we cannot rule out the possibility that maternal RNA or protein persist within the cardiac lineage of these mutants. Indeed, *trx*, *trr*, and *Set1* are expressed in early embryonic development prior to gastrulation which may provide residual activity in the mutants (Hallson et al., 2012; Prudencio et al., 2018; Sedkov et al., 1994). However, our study does identify and describe the importance of zygotic gene function of these genes in embryonic cardiac development. Recently, Zhu and colleagues (2023) have described specific roles for *trx*, *trr*, and *Set1* in the larval and adult *Drosophila* heart. Not only do the COMPASS-like HMTs regulate larval cardiac cell division by restricting over production of Tin CCs, but these genes also maintain global H3K4 methylation levels and myocardial gene expression necessary for normal cardiac physiology in the adult heart (Zhu et al., 2023).

Intriguingly, *trx* loss of function results in the complete loss of *Antp*, *abd-A*, and *Abd-B* expression within the dorsal vessel which in turn recapitulates the combined cardiac phenotypes of each individual *Hox* mutant (Lo et al., 2002; Lovato et al., 2002; Perrin et al., 2004; Ponzielli et al., 2002; Ryan et al., 2005). Previous work had identified *abd-A* as the primary selector gene for heart-proper development since its expression is both necessary and sufficient to drive heart-proper patterning within the dorsal vessel (Lo et al., 2002; Lovato et al., 2002; Perrin et al., 2004; Ponzielli et al., 2002). Thus, the most profound effect of *trx* inactivation is the loss of *abd-A* expression. It is this loss of *abd-A* expression that is responsible for the homeotic transformation of the heart-proper into a posterior aorta-like fate.

Second, the *trx* regulation of cardiac *Antp* and *Abd-B* expression plays important roles in defining the A/P patterning of the cardial cells along the dorsal vessel. At the A1/A2 segment boundary, *Antp* activity is required for the proper Svp cardiac cell progenitor divisions that produce a symmetric pair of two Svp CCs within the A2 hemisegment; hence the elimination of *Antp* expression in the dorsal vessel in *trx* mutant embryos results in only one Svp CC being produced in each A2 hemisegment. At the posterior end of the heart-proper, *Abd-B* expression suppresses excessive Tin CCs, thereby ensuring the production of a partial contralateral hemisegment consisting of two Svp CCs followed by two Tin CCs each (Lo et al., 2002; Lovato et al., 2002; Perrin et al., 2004). In *trx* mutant hearts, in the absence of *Abd-B* expression, this suppression of excessive Tin CCs in the A8 hemisegments is eliminated, resulting in supernumerary Tin CCs.

In contrast to the complete elimination of *Antp*, *abd-A*, and *Abd-B* expression in the dorsal vessel in *trx* mutant embryos, the loss of *trx* function merely alters the *Ubx* expression to a uniformly consistent level along the dorsal vessel. Our results thus suggest that in *trx* mutants, *Ubx*, by itself, may provide sufficient *Hox* activity to drive Svp CC specification. Within the wild-type embryo, *Ubx* expression is tightly controlled via multiple mechanisms involving auto-regulation, cross-regulation via other Hox genes, and several *cis*-regulatory elements (Bienz and Tremml, 1988; Karch et al., 1990; Macias et al., 1990; Maeda and Karch, 2006). Although the persistence of *Ubx* expression in *trx* mutants might initially suggest a regulatory mechanism that is not directly *trx-*dependent, we cannot rule out the possibility that *trx* may be directly responsible for the observed high levels of *Ubx* expression within wild-type A2-A4 cardiac hemisegments.

In conclusion, we have characterized the developmental functions of the *Drosophila* COMPASS-like H3K4 HMTs in heart development. Whereas *trx*, *trr*, and *Set1* all participate in the regulation of cardiac cell division, *trx* is also necessary for collinear *Hox* expression and the A/P patterning of the dorsal vessel. Our data suggest an evolutionarily conserved role for *trx* regulation of cardiac *Hox* expression in heart development. Given the importance of *Hox* activity in heart development and our findings in this report, we propose that the *trx* ortholog *Kmt2b* may play critical roles in cardiac *Hox* gene expression during mammalian heart development.

## MATERIALS AND METHODS

### *Drosophila* strains and genetics

The following mutant alleles and transgenes were obtained from the Bloomington *Drosophila* Stock Center: *trx^E2^* [FlyBase ID: FBal0017174] (Kennison and Tamkun, 1988), *trr^B^* [FlyBase ID: FBal0323346] (Kanda et al., 2013), *trr^C2375X^* [FBal0323346] (Kanda et al., 2013), *Set1^G12^* [FlyBase ID: FBal0265880] (Hallson et al., 2012), *Set1^G5^* [Flybase ID: FBal0265881] (Hallson et al., 2012), *Df(3R)BSC470,* designated *Df(trx)* in text, [Flybase ID: Fbal0265881] (Cook et al., 2012). The *trx^B11^* strain [FBal0032908] was provided by J. A. Kennison (Mazo et al., 1990). The *trx* and *Set1* alleles were maintained over *TM3, ftz-lacZ* balancers and *trr* alleles were maintained over *FM7c, ftz-lacZ* X-chromosome balancers before self-crossing. The embryos were genotyped by the absence of anti-β-galactosidase immunostaining of the expected *ftz-lacZ* balancer expression pattern. For validation of the *trx^E2^* and *Set1^G12^* mutant alleles, complementation tests were utilized. The *trx^E2^* allele failed to complement the *Df(3R)BSC470* deficiency, henceforth described as *Df(trx)*, and the *Set1^G12^* allele failed to complement the *Set1^G5^* allele. All genotypes used for analysis of cell division defects, *wg* expression, *Mp* expression, or *Hox* gene expression are described below.

**Table:** 

Oregon-R SNP iso2A (wild type)	Figs 1, 3A-B″,E-BB″,EE″-5, Figs S1 and S4
w^1118^ (wild type)	Fig. 3C-D″,CC-DD″
trx^B11^/+	Figs S1 and S5-S7
trx^E2^/+	Figs S1 and S5-S7
trx^E2^/trx^E2^	Figs 1–5, Figs S1, S2 and S4
trx^E2^/trx^B11^	Figs S1 and S5-S7
trx^E2^/Df(trx)	Figs S1 and S5-S7
trr^B^/Y	Fig. 1, Figs S1, S2 and S4-S3
trr^C2375X^/Y	Fig. S1
Set1^G12^/Set1^G12^	Fig. 1, Figs S1, S2 and S4-S3
Set1^G5^/Set1^G5^	Fig. S1

### Microscopy

Embryo fixation and fluorescent immunohistochemistry were performed as described previously examining protein expression (Ahmad et al., 2016, 2014). However, for Svp and Wg protein immunostaining, heat fixation with embryo wash buffer was utilized. Briefly, embryos were boiled in embryo wash buffer (140 mM NaCl, 0.03% Triton-X 100) for 10 s and immediately cooled with 4°C embryo wash buffer before devitellinization in 50:50 mixture of heptane and methanol followed by storage in methanol before staining. The following primary antibodies were used: guinea pig anti-H15 (NMR1, 1:1000, gift from J. B. Skeath) (Leal et al., 2009), rabbit anti-Mef2 [1:1000, Clone ID: Mef2 from Developmental Studies Hybridoma Bank (DSHB) and gift from J. Jacobs], chicken anti-β-galactosidase [1:500, catalog no. ab9361 (RRID:AB_307210) from Abcam, Inc], mouse anti-Svp (1:5, 5B11 from DSHB), rat anti-Mp (1:500, gift from T. Volk) (Harpaz et al., 2013), mouse anti-Ubx (1:10, Clone ID: FP3.38 from DSHB), mouse anti-Abd-A [1:100, Clone C-11 (sc-390990) from Santa Cruz Biotechnology, Inc.], mouse anti-Antp (1:20, Clone ID: 8C11 from DSHB), mouse anti-Wg (1:10, Clone ID 4D4 from DSHB), mouse anti-Slit (1:10, Clone ID: C555.6D from DSHB), and mouse anti-Abd-B (1:12.5, Clone ID: 1A2E9 from DSHB). Fluorescent microscopy was performed on a Zeiss AxioImager with Apotome. Z-stacks of stage 16 embryonic hearts were scanned at 20X with 0.60 µm intervals for anti-Mef2, anti-H15, anti-Svp, anti-Wg, anti-Slit, and anti-Hox immunostaining. Anti-Mp immunostaining was scanned at 40X with 0.31 µm intervals.

### Lumen width measurements

The Zen software suite of the Zeiss AxioImager microscope was used to measure the internal lumen diameter at five specific points along the dorsal vessel for Z-stacks of each embryo. The lumen width of the posterior aorta of a particular embryo was calculated as the mean of the internal lumen diameters measured across the middle of the four pairs of Tin CCs in the A2, A3, and A4 segments. The lumen width of the heart proper region of a specific embryo was the mean of the internal lumen diameters measured across the middle of the four pairs of Tin CCs in the A6 and A7 segments (Fig. S3).

### Statistical methods

A comparison of cell division errors between genotypes using Fisher exact tests is not appropriate as the hemisegments for a given embryo are correlated in their likelihood of having such errors (Ahmad et al., 2012). Comparison of cell division error rates between genotypes was thus done using regression models with the response variable being the proportion of hemisegment errors for each embryo. Due to violation of regression assumptions, e.g. non-normality and heteroscedasticity, permutation (randomization) tests were used to obtain reliable *P*-values (Manly, 2007).

For comparing rates between two genotypes, for example *trr^B^* and wild type, the following general linear model was used:

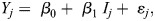
where *Y*_*j*_ is the proportion of hemisegment errors for embryo j and indicator variable *I*_*j*_ is 1 if embryo j has phenotype *trr^B^* and 0 otherwise. To obtain a permutation *P*-value for testing *H*_0_: *β*_1_=0, the estimate of *β*_1_ for the actual data is compared with the estimates obtained when the genotypes of the embryos are permuted, i.e. the phenotype labels are randomly shuffled among the embryos in the sample. The permutation test *P*-value is then *p*=(*n*+1)/(*N*+1) where *n* is the number of permutation estimates for *β*_1_, which exceed the estimate for the actual data and *N* is the number of permutations (Phipson and Smyth, 2010). In order to obtain highly reproducible *P*-values, *N*=10^6^ permutations were used for all permutation tests. All permutation tests were performed using R, version 4.2.2 (R Core Team, 2022).

For quantitation of Tin CC and Svp CC number changes in A2 and A8 hemisegments of wild type and *trx^E2^* mutant embryos, two-tailed, two-sample unequal variance *t*-tests were used with significance level of 0.05 (SPSS Statistics software; IBM; Version 29). For comparisons of Tin CC and Svp CC number changes in A2 and A8 hemisegments among multiple genotypes [wild type, *trx^E2^/+*, *trx^B11^/+*, *trx^E2^/trx^B11^*, and *trx^E2^/Df(trx)*], a one-way ANOVA was completed with SPSS BCa 5000 sample bootstrapping to assess between group statistical differences and significance for hemisegment specific differences.

ANOVAs with a significance level of 0.05 (SPSS Statistics software; IBM; Version 29) were used to compare the number of CCs immediately anterior to the Svp CCs of the A2 hemisegments.

Two-tailed, two-sample unequal variance *t*-tests with significance level of 0.05 (GraphPad; Dotmatics) were used for comparing lumen widths of the posterior aorta and the heart proper regions of appropriate genotypes.

## Supplementary Material

10.1242/biolopen.061919_sup1 Supplementary information

Table S1. Tabulation of changes (if any) in Svp CC and Tin CC numbers for each hemisegment in the wild type, *trx^E2^/+* heterozygotes, *trx^B11^/+* heterozygotes, *trx^E2^* mutant, transheterozygous *trx^E2^/Df(trx)* mutant, transheterozygous *trx^E2^/trx^B11^* mutant, hemizygous *trr^B^/Y* mutant, hemizygous *trr^C2375X^/Y* mutant, *Set1^G12^* mutant, and *Set1^G5^* mutant embryos used in this work. Note that each hemisegment is uniquely identified based on the genotype, specific embryo used, the particular row (left or right) of the dorsal vessel, and the hemisegment identity (A2, A3, A4, A5, A6, A7, or A8).
