# Supplementary material for: trithorax is an essential regulator of cardiac Hox gene expression and anterior-posterior patterning of the Drosophila embryonic heart tube
Source: Biol Open. 2025 Apr 2;14(4):bio061919. doi: 10.1242/bio.061919 (PMC11993250; doi:10.1242/bio.061919)
Supplement: Supplementary information [file biolopen-14-061919-s1.pdf]

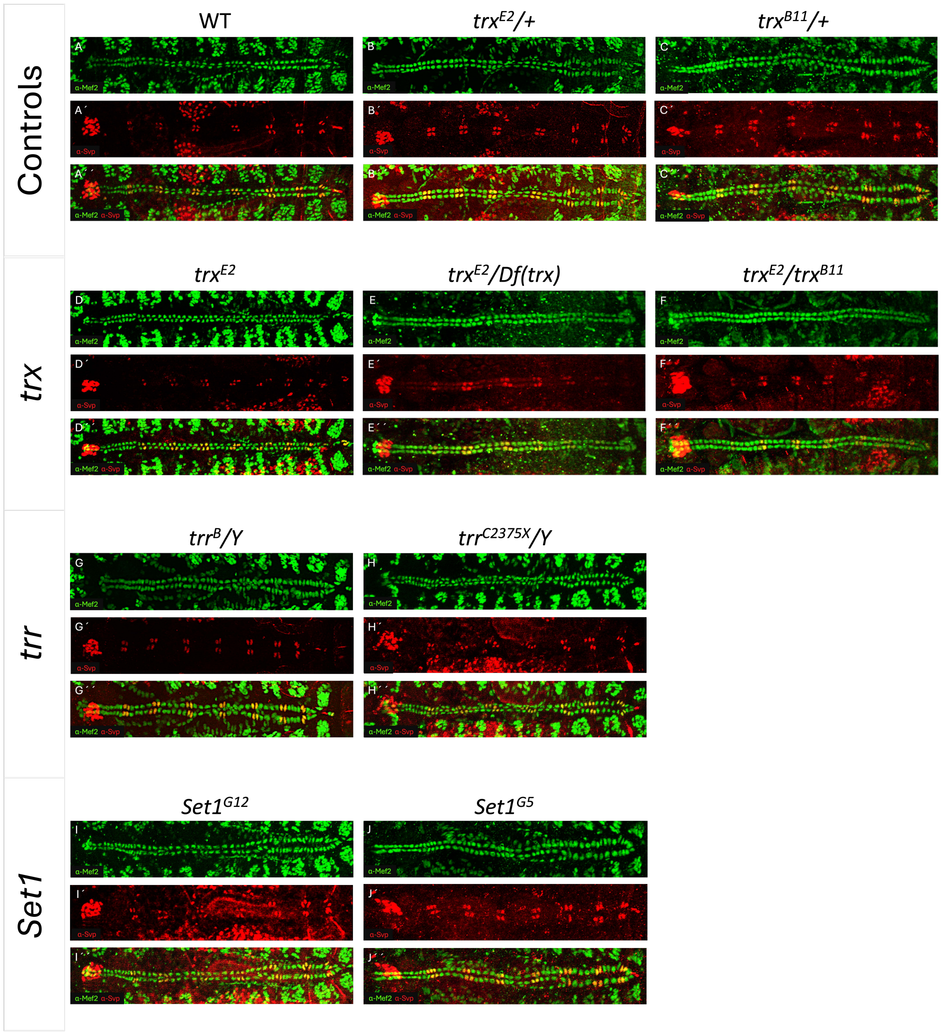

Fig. S1. Cardiac cell division phenotypes of H3K4 HMT mutant dorsal vessels. (A-J´´) Mef2 (green) and Svp (red) immunostaining displayed separately and in merged micrographs for representative dorsal vessels from controls: wild type (A-A´´), *trx*^{E2}/+ heterozygote (B-B´´), and *trx*^{B11}/+ heterozygote (C-C´´); *trx* loss of function embryos: *trx*^{E2} homozygote (D-D´´), *trx*^{E2}*/Df(trx)* transheterozygote (E-E´´), and *trx*^{E2}*/trx*^{B11} transheterozygote (F-F´´); *trr* loss of function embryos: *trr*^{B}*/Y* hemizygote (G-G´´) and *trr*^{C2375X}*/Y* hemizygote (H-H´´); and *Set1* loss of function embryos: *Set1*^{G12} homozygote (I-I´´) and *Set1*^{G5} homozygote (J-J´´).

Biology Open • Supplementary information

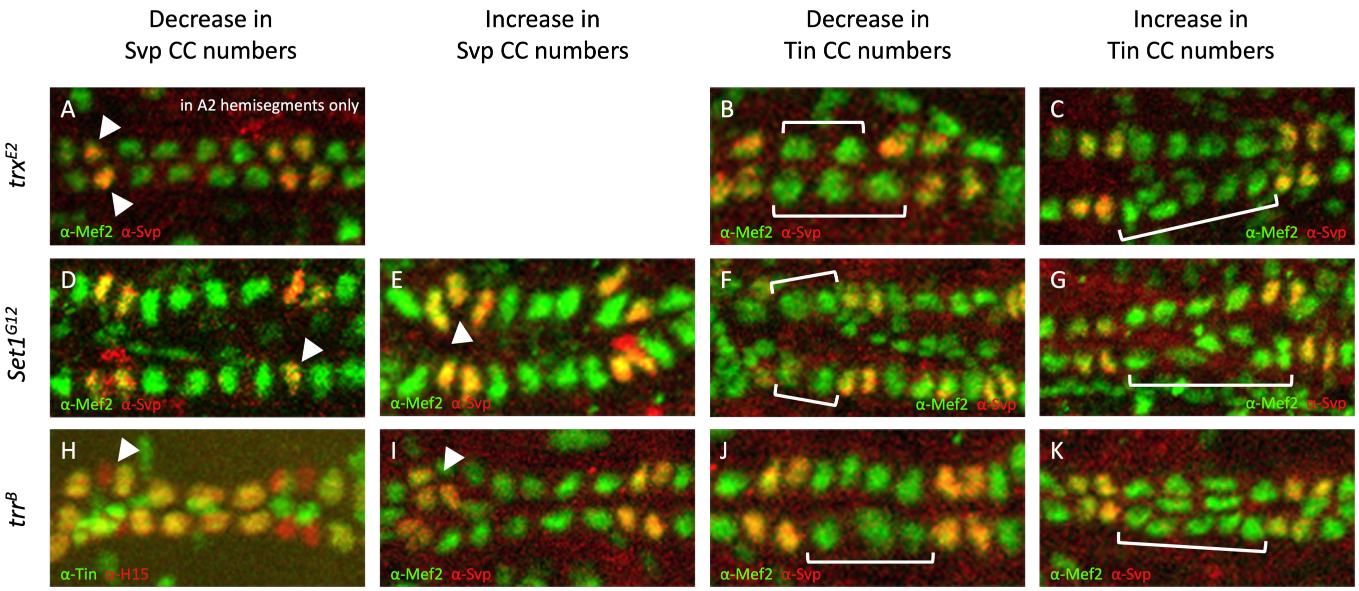

Fig. S2. Representative segments from H3K4 HMT mutant dorsal vessels exhibiting changes in Svp CC or Tin CC numbers. (A-K) Changes in Svp CC numbers (arrowheads) and Tin CC number (brackets) in *trx*^{E2} (A-C), *Set1*^{G12} (D-G), and *trr*^{B} (H-K) mutants. (A) Note that in *trx* mutants, while Svp CC numbers are consistently altered (reduced to one per hemisegment) always in A2 hemisegments, no significant changes in Svp CC numbers are detected in the other hemisegments. (B-C) Both decreases (B) and increases (C) in Tin CC numbers are seen in A2-A8 hemisegments for *trx* mutants. (D-K) Increases and decreases in both Svp CC numbers and Tin CC numbers are seen for both *Set1* and *trr* mutants. Panel H shows segments from a *trr* mutant dorsal vessel immunostained with both anti-H15 (red) and anti-Tin (green). Consequently, Svp CCs are labeled red and Tin CCs are labeled yellow in this panel (H). In every other panel (A-G, I-K) dorsal vessels are immunostained with anti-Svp (red) and anti-Mef2 (green). Thus, in those panels, Svp CCs are labeled yellow and Tin CCs are labeled green.

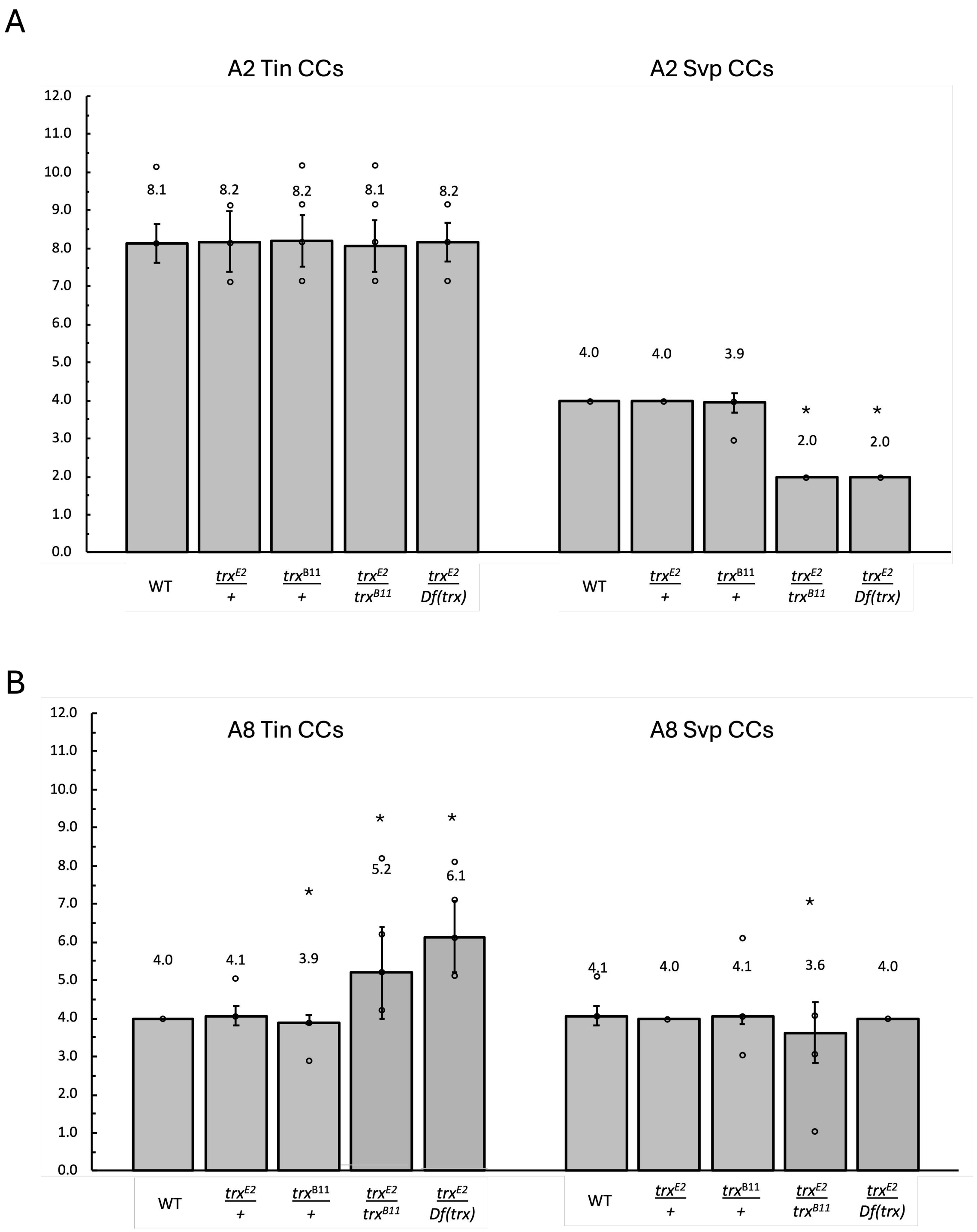
A
A2 Tin CCs
A2 Svp CCs
B
A8 Tin CCs
A8 Svp CCs
WT
trxE2 / +
trxB11 / +
trxE2 / trxB11
trxE2 / Df(trx)

Fig. S3. Quantitation of cardial cell (CC) numbers of A2 and A8 segments in wild type, heterozygous, and transheterozygous *trx* mutant embryos. Comparison of Tin CC and Svp CC numbers in A2 and A8 cardiac segments in wild type (n=15), heterozygous $trx^{E2}/+$ (n=17), heterozygous $trx^{B11}/+$ (n=14), transheterozygous trx^{E2}/trx^{B11} mutant (n=17), and transheterozygous trx^{E2}*/Df(trx)* mutant (n=17). (A) Each wild type A2 segment, comprised of two contralateral A2 hemisegments, consists of approximately 8 Tin CCs and 4 Svp CCs (i.e. 4 Tin CCs and 2 Svp CCs per hemisegment). The number of Svp CCs within A2 hemisegments are reduced to 2 Svp CCs within the transheterozygous *trx* mutants in contrast to the wildtype and heterozygous controls, however, the A2 Tin CC number is not affected. These results replicate a defect in the cardiac progenitor cell division process that gives rise to two Svp-CCs in the A2 hemisegments in trx^{E2} mutant. (B) Each wild type A8 segment consists of approximately 4 Tin CCs and 4 Svp-CCs (i.e., each of the two posteriormost contralateral A8 hemisegments comprising this segment have 2 Tin-CCs and 2 Svp-CCs). While the number of Svp-CCs in the A8 segment is not increased in the transheterozygous *trx* mutants compared to that in the wild type or *trx* heterozygous embryos, the number of Tin-CCs is significantly increased within the transheterozygous *trx* mutants compared to that in wild type or *trx* heterozygous embryos in a manner similar to the trx^{E2} mutant. Therefore, *trx* restricts Tin CC progenitor differentiation in the A8 segment. Mean CC numbers ± s.d. are reported. A one way ANOVA was completed with SPSS BCa 5000 sample bootstrapping to assess between group statistical differences and significance for hemisegment specific differences. Asterisks (*) associated with trx^{E2}/trx^{B11} quantitation indicate significant (P<0.05) differences in hemisegment specific cell numbers between trx^{E2}/trx^{B11} and wild type, trx^{E2}/trx^{B11} and $trx^{E2}/+$, and trx^{E2}/trx^{B11} and $trx^{B11}/+$. Asterisks (*) associated with trx^{E2}*/Df(trx)* quantitation indicate significant (P<0.05) differences in hemisegment specific cell numbers between trx^{E2}*/Df(trx)* and wild type and trx^{E2}*/Df(trx)* and $trx^{B11}/+$.

Biology Open • Supplementary information

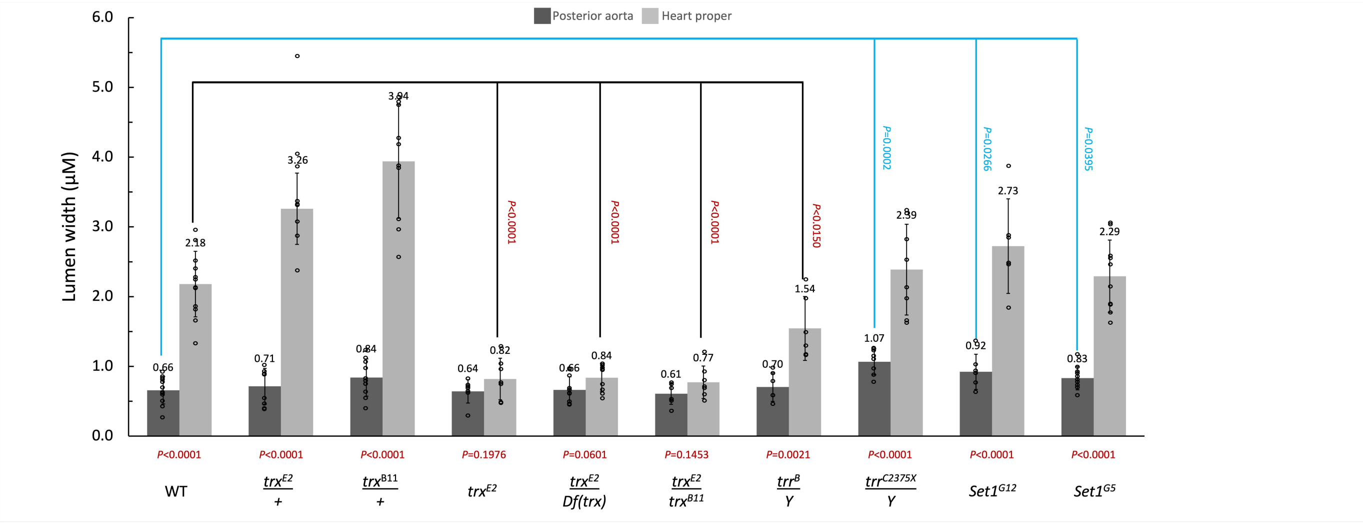

Fig. S4. Quantitation of lumen widths in the posterior aorta and the heart-proper regions of the dorsal vessels of wild type, *trx* mutant, *trr* mutant, and *Set1* mutant embryos. (A) Comparison of lumen widths between the posterior aorta and the heart-proper regions of wild type (n=12), trx^{E2}/+ heterozygotes (n=9), trx^{B11}/+ heterozygotes (n=10), trx^{E2} mutant (n=7), transheterozygous trx^{E2}/*Df(trx)* mutant (n=10), transheterozygous trx^{E2}/trx^{B11} mutant (n=7), hemizygous trr^{B}/*Y* mutant (n=6), hemizygous trr^{B}/*Y* mutant (n=8), $Set1^{G12}$ mutant (n=6), and $Set1^{G5}$ mutant (n=10) embryos at stage 16. Mean internal widths ± s.d. of the lumen at the posterior aorta are reported. Two-tailed, two-sample unequal variance t-tests with significance level of 0.05 were used for comparing lumen widths. Within the wildtype, heterozygous *trx*, *trr* mutant, and *Set1* mutant embryos, significant differences in lumen widths are found between the posterior aorta and the heart-proper regions. No such significant difference is detected in the width of the lumen between the posterior aorta and the heart-proper region in embryos of the genotypes trx^{E2}, trx^{E2}/*Df(trx)*, or trx^{E2}/trx^{B11}. (B-E) Representative dorsal vessels from wild type, trx^{E2} mutant, trr^{B} mutant, and $Set1^{G12}$ mutant embryos illustrating how lumen width was calculated. The lumen width of the posterior aorta of a particular embryo was calculated as the mean of the widths (white lines) measured with the Zeiss AxioImager across the middle of the four pairs of Tin CCs in the A2, A3, and A4 segments. The lumen width of the heart-proper region (bracket) for any specific embryo was the mean of the widths (white lines) across the middle of the four pairs of Tin CCs in the A6 and A7 segments.

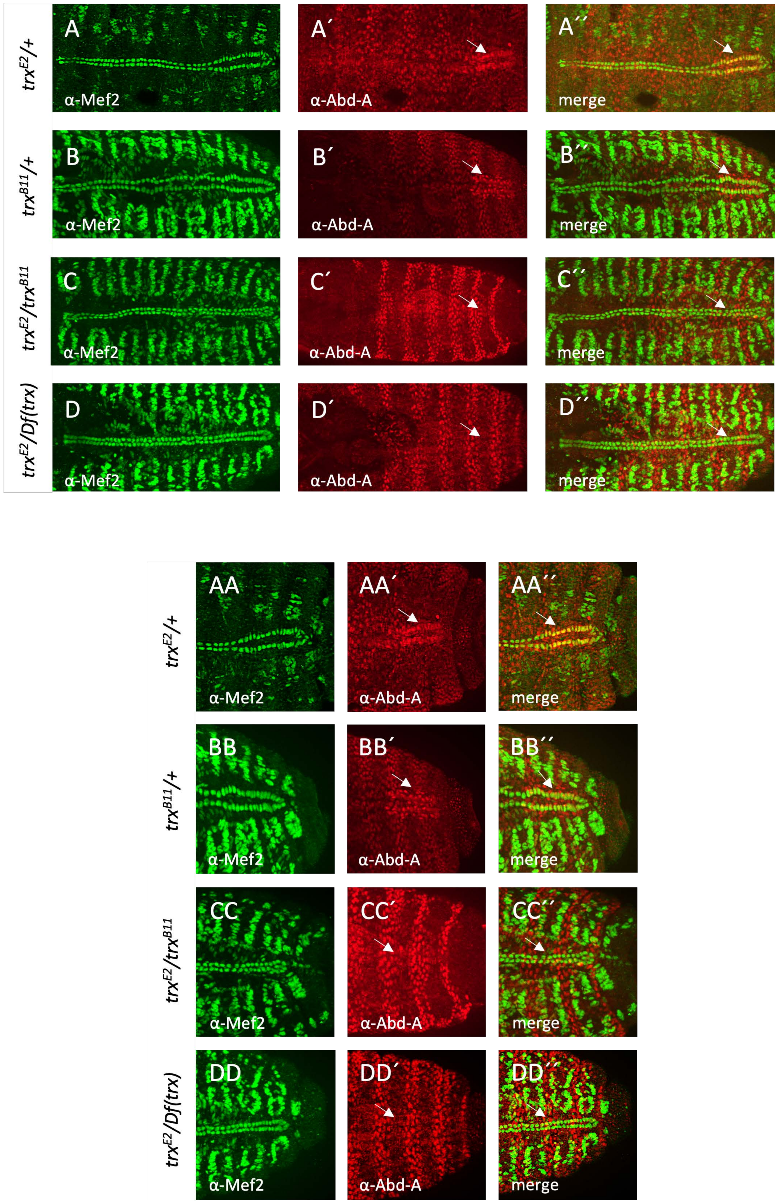

Fig. S5. Loss of heart-proper-specific *abd-A* expression within transheterozygous *trx* mutants. *Abd-A* expression (red) within dorsal vessels from embryos with different genotypic combinations of *trx* mutant alleles and a *trx*-excising deficiency is detected by antibody staining. All CCs of the dorsal vessel are identified using anti-Mef2 (green). (A-DD´´) The posterior regions of the heterozygous trx^{E2}/+ (A´ and AA´, arrow; n= 4, 4/4 embryos) and heterozygous trx^{B11}/+ (B´ and BB´; arrow, n= 4, 4/4 embryos) embryos display strong Abd-A immunostaining and the characteristic morphology of heart-proper, while the posterior dorsal vessels of the transheterozygous *trx* mutants: trx^{E2}/trx^{B11} (C´ and CC´, arrow; n=8, 8/8 embryos) and trx^{E2}/*Df(trx)* (D´ and DD´, arrow; n=4, 4/4 embryos) lack both Abd-A staining and heart-proper morphology. Note that (AA-DD´´) are higher magnification views of the posterior dorsal vessels in (A-D´´). These results indicate that the transheterozygous *trx* mutants phenocopy the trx^{E2} cardiac defects.

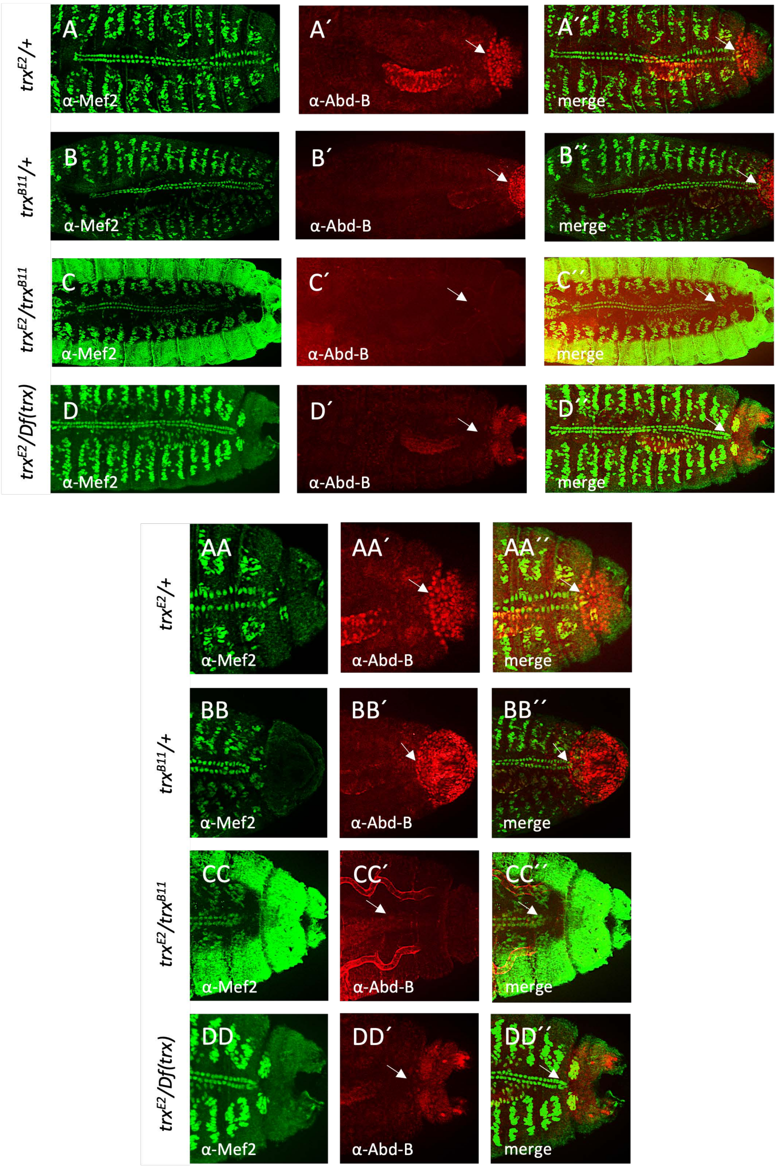

Fig. S6. Loss of heart-proper-specific *abd-B* gene expression within transheterozygous *trx* mutants. *Abd-B* expression (red) within dorsal vessels from embryos with different genotypic combinations of *trx* mutant alleles and a *trx*-excising deficiency is detected by antibody staining. All CCs of the dorsal vessel are identified using anti-Mef2 (green). (A-DD´´) The posterior-most regions of the heterozygous *trx*^{E2}/+ (A´ and AA´, arrow; n= 3, 3/3 embryos) and heterozygous *trx*^{B11}/+ (B´ and BB´; arrow, n= 3, 3/3 embryos) dorsal vessels display Abd-B immunostaining within the A8 CCs, while the posterior dorsal vessels of the transheterozygous *trx* mutants: *trx*^{E2}/*trx*^{B11} (C´ and CC´, arrow; n=5, 5/5 embryos) and *trx*^{E2}/*Df(trx)* (D´ and DD´, arrow; n=4, 4/4 embryos) lack any Abd-B staining. Note that (AA-DD´´) are higher magnification views of the posterior dorsal vessels in (A-D´´).

Biology Open • Supplementary information

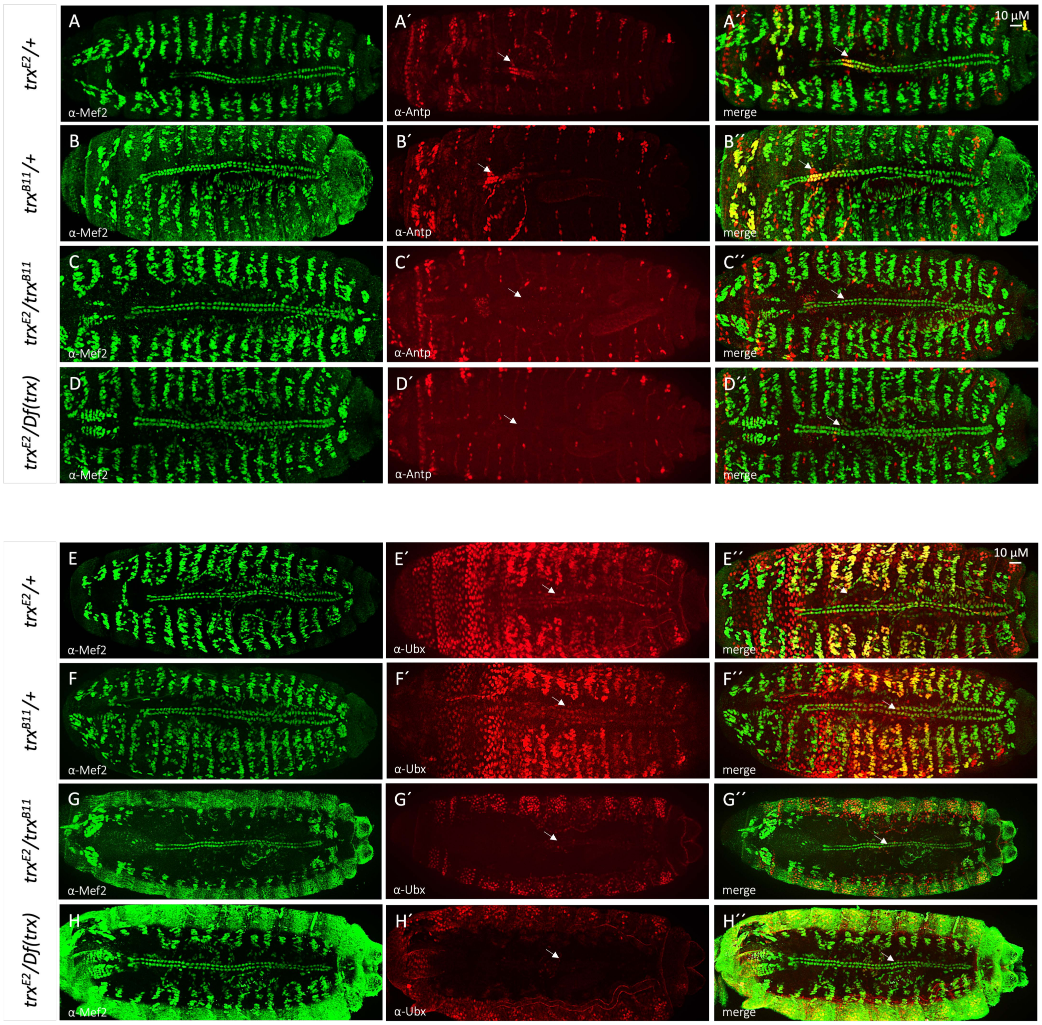

Fig. S7. *Antp* and *Ubx* expression within transheterozygous *trx* mutants. *Antp* and *Ubx* expression domains in wild type and *trx* mutant embryos are detected by immunostaining with the appropriate antibodies (red), while all CCs of the dorsal vessel are identified using the anti-Mef2 antibody (green). (A-D´´) Antp protein marks the boundary between the anterior and posterior aorta. (A-B´´) High *Antp* expression within the heterozygous trx^{E2}/+ (A´ and AA´, arrow; n= 2, 2/2 embryos) and heterozygous trx^{B11}/+ (B´ and BB´; arrow, n= 3, 3/3 embryos) dorsal vessels. (C-D´´) In contrast, *Antp* expression is lost within the aorta of the transheterozygous *trx* mutants: trx^{E2}/trx^{B11} (C´ and C´´, arrow; n=2, 2/2 embryos) and trx^{E2}/*Df(trx)* (D´ and D´´, arrow; n=3, 3/3 embryos). (E-H´´) Ubx protein is broadly localized across the posterior aorta. (E-F´´) *Ubx* is expressed at different levels along the dorsal vessel being highest across the A2-A4 hemisegments of the posterior aorta in the heterozygous *trx* embryos: heterozygous trx^{E2}/+ (E-E´´, arrow; n= 3, 3/3 embryos) and heterozygous trx^{B11}/+ (F´ and FF´´; arrow, n= 2, 2/2 embryos). (G-H) Ubx levels are significantly reduced within the posterior heart tubes (arrow) of the transheterozygous *trx* mutants: trx^{E2}/trx^{B11} (G´ and G´´, arrow; n=2, 2/2 embryos) and trx^{E2}/*Df(trx)* (H´ and H´´, arrow; n=2, 2/2 embryos).

Biology Open • Supplementary information

Table S1. Tabulation of changes (if any) in Svp CC and Tin CC numbers for each hemisegment in the wild type, *trx^{E2}/+* heterozygotes, *trx^{B11}/+* heterozygotes, *trx^{E2}* mutant, transheterozygous *trx^{E2}/Df(trx)* mutant, transheterozygous *trx^{E2}/trx^{B11}* mutant, hemizygous *trr^{B}/Y* mutant, hemizygous *trr^{C2375X}/Y* mutant, *Set1^{G12}* mutant, and *Set1^{G5}* mutant embryos used in this work. Note that each hemisegment is uniquely identified based on the genotype, specific embryo used, the particular row (left or right) of the dorsal vessel, and the hemisegment identity (A2, A3, A4, A5, A6, A7, or A8).

Available for download at
https://journals.biologists.com/bio/article-lookup/doi/10.1242/bio.061919#supplementary-data
